# Histopathological Response After Neoadjuvant Chemotherapy for High-Risk Soft-Tissue Sarcomas: A Secondary Analysis of a Randomized Clinical Trial

**DOI:** 10.1001/jamanetworkopen.2025.40177

**Published:** 2025-11-06

**Authors:** Sandro Pasquali, Paola Collini, Cleofe Romagosa, Jean-Michel Coindre, Sara Pizzamiglio, Paolo Verderio, Valeria Duroni, Marta Barisella, Emanuela Palmerini, Vittorio Quagliuolo, Javier Martin Broto, Antonio Lopez Pousa, Giovanni Grignani, Antonella Brunello, Jean-Yves Blay, Iwona Lugowska, Valeria Fontana, Giuseppe Bianchi, Elena Palassini, Salvatore Lorenzo Renne, Paolo Giovanni Casali, Rosalba Miceli, Marta Sbaraglia, Marco Gambarotti, Silvia Bagué, Angelo Paolo Dei Tos, Silvia Stacchiotti, Alessandro Gronchi

**Affiliations:** 1 Molecular Pharmacology, Department of Experimental Oncology, Fondazione Istituto di Ricovero e Cura a Carattere Scientifico (IRCCS) Istituto Nazionale dei Tumori di Milano, Milano, Italy; 2 Soft Tissue Tumor Pathology Unit, Department of Advanced Diagnostics, Fondazione IRCCS Istituto Nazionale dei Tumori di Milano, Milano, Italy; 3 Pathology Department, Vall d’Hebron University Hospital, Barcelona, Spain; 4 Department of Pathology, Institut Bergonié, Bordeaux, France; 5 Institut National de la Santé et de la Recherche Médicale (INSERM) U1218 ACTION, Institut Bergonié, Bordeaux, France; 6 Unit of Bioinformatics and Biostatistics, Fondazione IRCCS Istituto Nazionale dei Tumori, Milan, Italy; 7 Pathology Unit, Azienda SocioSanitaria Territoriale (ASST) Fatebenefratelli Sacco, Milan, Italy; 8 Osteoncology, Bone and Soft Tissue Sarcomas and Innovative Therapies Unit IRCCS Istituto Ortopedico Rizzoli, Bologna, Italy; 9 Miller School of Medicine, University of Miami, Miami, Florida; 10 Surgery Department, IRCCS Humanitas Research Hospital, Rozzano, Italy; 11 Oncology Department, Fundación Jiménez Díaz University Hospital, Madrid, Spain; 12 Medical Oncology Department, Hospital de la Santa Creu i Sant Pau, Carrer de Sant Quintí, Barcelona, Spain; 13 Medical Oncology Unit, Città della Salute e della Scienza Hospital, Turin, Italy; 14 Department of Oncology, Medical Oncology 1 Unit, Istituto Oncologico Veneto IOV IRCCS, Padova, Italy; 15 Centre Léon Bérard & Université Claude Bernard Lyon 1, Lyon, France; 16 Department of Soft Tissue/Bone Sarcoma and Melanoma, Centrum Onkologii, Instytutim, Marii Sklodowskiej-Curie, Warsaw, Poland; 17 Clinical Trial Center and Department of Epidemiology, IRCCS Ospedale Policlinico San Martino, IST Istituto Nazionale per la Ricerca sul Cancro, Genoa, Italy; 18 Orthopedic Oncology Unit, IRCCS Istituto Ortopedico Rizzoli, Bologna, Italy; 19 Department of Cancer Medicine, Fondazione IRCCS Istituto Nazionale dei Tumori di Milano, Milano, Italy; 20 Department of Pathology, IRCCS Humanitas Research Hospital, Rozzano, Milan, Italy; 21 Surgical Pathology and Cytopathology Unit, Department of Medicine–DIMED, University of Padua, Padua, Italy; 22 Department of Data Science, Fondazione IRCCS Istituto Nazionale dei Tumori di Milano, Milano, Italy; 23 Department of Pathology, IRCCS Istituto Ortopedico Rizzoli, Bologna, Italy; 24 Pathology Department, Hospital de la Santa Creu i Sant Pau, Universitat Autònoma de Barcelona, Barcelona, Spain; 25 Sarcoma Service, Department of Surgery, Fondazione IRCCS Istituto Nazionale dei Tumori di Milano, Milano, Italy

## Abstract

**Importance:**

Treatment of high-risk soft-tissue sarcoma (STS) of extremity or trunk wall involves neoadjuvant chemotherapy (NACT) followed by surgery. Histopathological response could estimate patient outcomes.

**Objective:**

To characterize morphological changes in surgical specimens of patients treated with NACT with or without radiotherapy (RT) to identify histopathological features that stratify risk of recurrence and ultimately estimate the benefit from neoadjuvant treatments.

**Design, Setting, and Participants:**

This was a preplanned prospective secondary analysis of the ISG-STS 1001 clinical trial, a study with both a randomized clinical trial (conducted between 2011 and 2016) and a nonrandomized patient cohort (included between 2016 and 2020) at 32 centers across Italy, Spain, France, and Poland. Participants were patients with STS randomly assigned to receive either anthracycline plus ifosfamide or histotype-tailored (also termed histology tailored) NACT. Data analyses were performed from January to June 2023.

**Intervention:**

Participants received 3 cycles of anthracycline plus ifosfamide or histotype-tailored NACT with or without RT followed by surgery.

**Main Outcomes and Measures:**

The primary outcome was disease-free survival (DFS). Histopathological features considered included the proportion of stainable tumor cells, tumor necrosis, hemorrhage, fibrohistiocytic reaction with hemosiderin, sclerosis or fibrosis, and sclerohyalinosis. The proportion of stainable tumor cells was classified according to the European Organization for Research and Treatment of Cancer–Soft Tissue and Bone Sarcoma Group categories or as absent or present. The continuous variable of sclerohyalinosis, expressed as a percentage, was categorized based on the second tertile of its distribution (20%). Tumor necrosis, hemorrhage, fibrohistiocytic reaction with hemosiderin, sclerosis or fibrosis, which were also expressed as a percentage, were classified as absent or present.

**Results:**

A total of 388 patients (201 in randomized cohort, 187 in nonrandomized cohort; median [IQR] age, 50 [41-60] years; 245 males [63.1%]) were evaluable for histopathological response. In the randomized cohort, after a median (IQR) follow-up of 86 (70-99) months, 115 of 201 patients (57.2%) developed a disease recurrence. The proportion of stainable tumor cells (>1%) was not associated with DFS (hazard ratio [HR], 1.47; 95% CI, 0.36-5.98; *P* = .59). Necrosis (>1%) was associated with shorter DFS (HR, 3.11; 95% CI, 1.36-7.14; *P* = .007), while sclerohyalinosis greater than 20% was associated with longer DFS (HR, 0.51; 95% CI, 0.28-0.94; *P* = .03). Exclusion of patients who received preoperative RT did not alter these associations. In patients randomly assigned to anthracycline plus ifosfamide (n = 98), sclerohyalinosis greater than 20% remained associated with longer DFS (HR, 0.24; 95% CI, 0.09-0.67; *P* = .007). These findings were confirmed when a broader cohort (n = 187) was included.

**Conclusions and Relevance:**

In this secondary analysis of a randomized clinical trial, the proportion of stainable tumor cells, currently considered as the most relevant posttreatment change, did not stratify patient risk. The findings support consideration of the presence of sclerohyalinosis (>20%) to identify patients with the best outcome after NACT.

## Key Points

**Question:**

Does histopathological response after neoadjuvant chemotherapy (NACT) estimate outcomes in patients with high-risk soft-tissue sarcoma (STS) of extremity or trunk wall?

**Findings:**

In this preplanned secondary analysis of a clinical trial involving 388 patients, the proportion of stainable tumor cells was not associated with disease-free survival (DFS). Necrosis was associated with worse DFS, and presence of sclerohyalinosis greater than 20% estimated improved DFS.

**Meaning:**

These findings suggest that the presence of sclerohyalinosis may serve as a novel histopathological marker of favorable response to NACT in STS, challenging the current emphasis on the proportion of stainable tumor cells and informing patient risk stratification and treatment evaluation.

## Teaser

This secondary analysis of a randomized clinical trial evaluates the histopathological features associated with risk of recurrence that can be used to estimate outcomes in patients with high-risk soft-tissue sarcoma of extremities or the trunk wall.

## Introduction

Histopathological response to neoadjuvant therapies, usually defined as presence of residual stainable tumor cells and other posttreatment changes, is considered a surrogate end point for identifying patients who benefit from these treatment strategies.^1,2,3,4^ Soft-tissue sarcomas (STS) are a rare group of malignant neoplasms in which surgery is the mainstay of treatment.^5^ Neoadjuvant chemotherapy (NACT) is considered when the risk of tumor metastasis is high to increase the chance of cure for patients, particularly in selected STS histotypes emerging in the extremity or trunk wall.^5^

The value of quantification of residual stainable tumor cells in estimating patient outcomes is well established for Ewing sarcoma of bone and osteosarcoma.^6,7^ Despite challenges in interpreting pathological responses in these tumors (eg, potential misclassification due to cutoff use), histopathological changes after neoadjuvant treatment remain among the most relevant factors in estimating patient outcomes. In STS, tumor necrosis represents an intrinsic feature of STS characterized by high malignant neoplasm grade,^8,9^ which is one of the major determinants of the indication for NACT.^10,11^ Morphological analysis cannot differentiate between tumor-intrinsic and treatment-induced necrosis, thus lowering the utility of this tumor characteristic for assessing treatment response in STS. Retrospective analysis from a clinical trial on NACT associated tumor response at magnetic resonance imaging (MRI) with stainable tumor cells, which defined a very good response when they were represented in less than 10% of the tumor. The European Organization for Research and Treatment of Cancer–Soft Tissue and Bone Sarcoma Group (EORTC-STBSG) included this information in a classification of histopathological tumor response after NACT. Consensus on the role of quantification of residual stainable tumor cells in posttreatment changes, including their cutoffs, is lacking. We hypothesized that NACT with or without radiotherapy (RT) induces histopathological changes to tumor tissue that could estimate patient outcomes. In this secondary analysis, we aimed to characterize morphological changes in surgical specimens of patients treated with NACT with or without RT to identify histopathological features that stratify risk of recurrence and ultimately estimate the benefit from neoadjuvant treatments.

## Methods

This preplanned prospective secondary analysis of the ISG-STS 1001 clinical trial (ClinicalTrials.gov Identifier: NCT01710176) was conducted from 2011 to 2020. Information about this trial, along with the study protocol, is described elsewhere^12,13,14,15^ and is available in Supplement 1. In brief, the ISG-STS 1001 trial, conducted at 32 centers across Italy, Spain, France, and Poland, included both a randomized clinical trial (between 2011 and 2016) and a nonrandomized patient cohort (between 2016 and 2020). The randomized trial was an open-label, phase 3 study that randomly assigned patients to receive either 3 cycles of anthracycline plus ifosfamide or histotype-tailored (also termed *histology tailored*) NACT followed by surgery. The appropriate independent ethics committee at each participating center approved the protocol and all amendments. The ISG-STS 1001 trial was conducted in accordance with the Declaration of Helsinki.^16^ All patients provided written informed consent before enrollment. We followed the Consolidated Standards of Reporting Trials (CONSORT) reporting guideline.

### Assessment of Histopathological Response

Histopathological analysis of posttreatment surgical specimens was performed, assessing all available slides. For each enrolling center, 1 local pathologist was responsible for sampling the surgical specimen, following the study protocol. This process was conducted in collaboration with the operating surgeon and, where possible, the radiologist, leading to a histopathological diagnosis. Subsequently, blinded national central reviewers (1 per participating country) were engaged to independently review all national cases and complete the corresponding forms. Whenever possible, cases underwent histological analysis during meetings that included both pathologists and radiologists, occurring concurrently with the radiological review. A lead investigator (P.C.) meticulously reviewed all submitted forms. In the event of discrepancies, cases were requested for reevaluation until a consensus was achieved.

**Figure 1.**
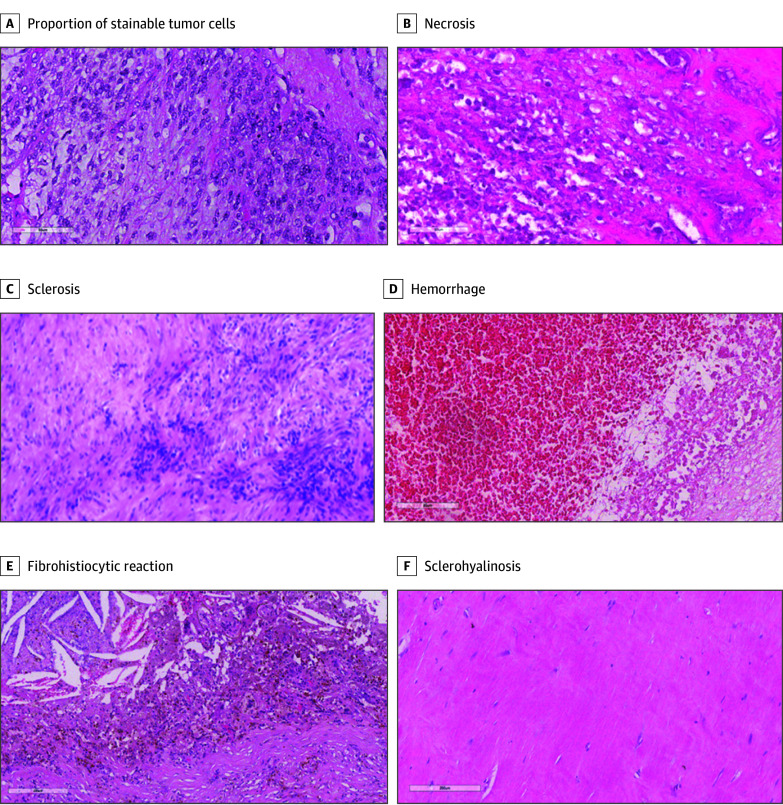
Features Considered to Characterize Histopathological Tumor Response in Trial Participants Treated With Neoadjuvant Chemotherapy With or Without Radiotherapy Histopathological images show residual stainable tumor cells (A) and posttreatment changes (B-F) in a surgical specimen of pretreated sarcoma (formalin-fixed paraffin-embedded tissue with hematoxylin-eosin stain magnification x200).

The following histopathological features were considered and scored a percentage relative to the whole tumor bed: proportion of stainable tumor cells in the tumor bed and each posttreatment change in the tumor bed, including tumor necrosis, hemorrhage, fibrohistiocytic reaction with hemosiderin, sclerosis or fibrosis, and sclerohyalinosis (Figure 1). The sum of the percentages was 100%. The occurrence of a cystic component was considered, especially for synovial sarcomas where posttreatment macroscopic diameter was shorter than that measured at preoperative radiological imaging. Stainable tumor cells were considered to be neoplastic cells that retained their structural integrity and properly absorbed hematoxylin and eosin staining, allowing for clear visualization of nuclear and cytoplasmic features. Stainable cells are characterized by distinctly visible nuclei under hematoxylin. The disappearance of this staining is a key diagnostic marker, serving as a histological sign of cell necrosis. Stainable tumor cells were quantified as a percentage that represents the proportion between the areas of stainable tumor cells and areas of the tumor.

Sclerosis or fibrosis was defined by the proliferation of fibroblasts and increased deposition of collagen fibers in the absence of a hyaline matrix. Histologically, it was presented as an expansion of the stromal component with interwoven or compact collagen bundles, often accompanied by spindle-shaped fibroblasts.

Sclerohyalinosis (eFigure 1 in Supplement 2) was defined by the presence of a dense, homogenized collagen with a paucicellular deposition of an eosinophilic hyaline matrix. Histologically, it exhibited a glassy, acellular appearance with sparse, atrophic or spindle-shaped fibroblasts embedded within the matrix. Scattered inflammatory cells—predominantly lymphocytes and macrophages—may be present, along with rare stainable tumor cells. The hyalinized stroma may show focal dystrophic calcifications, and in some cases, small, obliterated vascular structures can be observed.

### Statistical Analysis

Statistical analysis investigated the association between each characteristic of histopathological response and disease-free survival (DFS). DFS, which was the primary outcome of the ISG-STS 1001 trial, was defined as the time between surgery and the first recurrence or the last follow-up for nonrecurrence.

First, analyses were conducted in patients included in the randomized cohort. Second, analyses were restricted to patients who received NACT without preoperative RT and to patients who were randomly assigned to receive anthracycline plus ifosfamide, to reduce possible confounding effect of RT and histotype-tailored NACT, respectively. Third, the randomized and nonrandomized cohorts of the ISG-1001 trial were merged.

The proportion of stainable tumor cells was classified according to EORTC-STBSG categories or as absent or present. The continuous variable of sclerohyalinosis, expressed as a percentage, was categorized based on its distribution, and the second tertile was used as the cutoff point. Tumor necrosis, hemorrhage, fibrohistiocytic reaction with hemosiderin, and sclerosis or fibrosis, also expressed as a percentage, were classified as absent or present.

Detailed information about statistical tests used in this analysis are provided in the eMethods in Supplement 2. Data analyses were performed from January to June 2023 using SAS Studio, version 5.2 (SAS Institute Inc). Statistical significance was set at *P* = .05.

## Results

**Figure 2.**
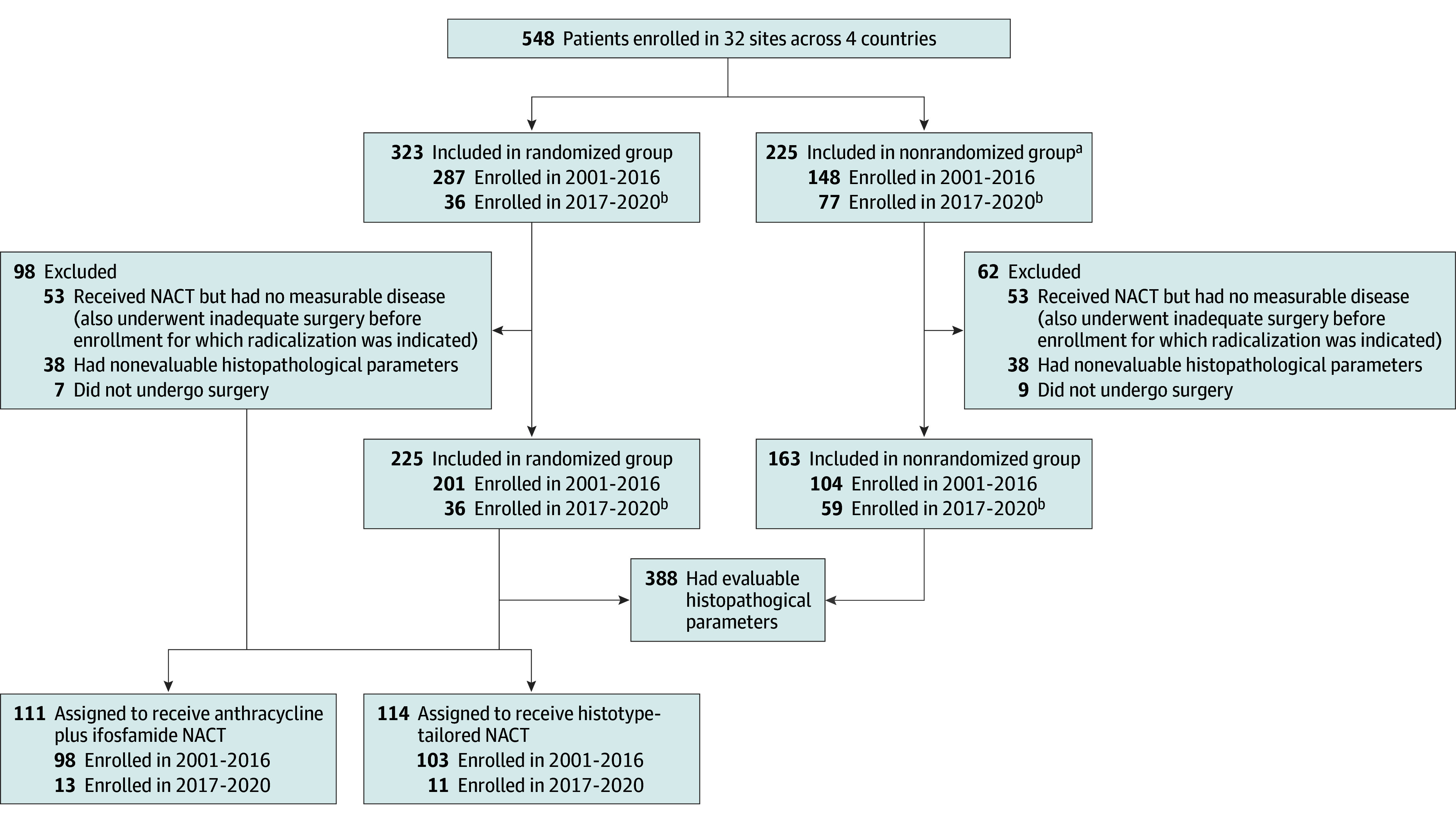
CONSORT Diagram of the ISG-1001 Clinical Trial NACT indicates neoadjuvant chemotherapy. ^a^Affected by histologies not randomized after central pathological review and treated with anthracycline plus ifosfamide. ^b^Includes classification of patients after protocol amendment 3.0 in January 2017.

Three hundred eighty-eight patients (median [IQR] age, 50 [41-60] years; 143 females [36.9%], 245 males [63.1%]), from a total of 548 enrolled in the ISG-STS 1001 clinical trial, had surgical specimens including a residual tumor evaluable for histopathological response (Figure 2, Table 1). Histopathological response was assessed in 201 (70.0%) of 287 patients in the randomized cohort. Among excluded patients, 47 had re-excision without macroscopic disease, 7 did not have surgery, and 32 had missing information on pathological response. Primary outcome of this trial is detailed in previous reports.^12,13^ In addition, the present analysis included 24 patients with myxoid liposarcoma who were enrolled in the expansion cohort of the randomized trial.^14^ In this analysis, we also evaluated data from 163 patients in the nonrandomized cohort. Patient and tumor characteristics are reported in Table 1.

**Table 1.** Clinical-Pathological Features of Patients With High-Risk Soft-Tissue Sarcomas of Extremity or Trunk Wall Treated With NACT With or Without Radiotherapy and Surgery in the ISG-1001 Clinical Trial

Characteristic	Patients, No. (%)				All (N = 388)
	Anthracycline plus ifosfamide NACT (n = 274)			Histotype-tailored NACT (n = 114)	
	All (n = 274)	Randomized (n = 111)	Nonrandomized (n = 163)	Randomized (n = 114)	
Age, y					
Mean (SD)	50.5 (12.0)	47.6 (11.2)	52.5 (12.2)	48.6 (13.4)	49.9 (12.5)
Median (IQR)	51 (41-60)	47 (39-57)	53 (44-62)	50 (40-60)	50.5 (41-60)
Sex					
Male	173 (63.1)	70 (63.1)	103 (63.2)	72 (63.2)	245 (63.1)
Female	101 (36.9)	41 (36.9)	60 (36.8)	42 (36.8)	143 (36.9)
Tumor size, mm					
Mean (SD)	119.1 (54.9)	114.1 (45.9)	122.5 (60.2)	111 (66.3)	116.7 (58.6)
Range	10-350	32-270	10-350	46-680	10-680
Median (IQR)	110 (80-149)	107 (83-135)	115 (80-152)	99.5 (75-140)	107 (80-142.5)
Histologic type					
High-grade myxoid liposarcoma	70 (25.6)	35 (31.5)	35 (21.5)	31 (27.2)	101 (26.0)
Synovial sarcoma	40 (14.6)	25 (22.5)	15 (9.2)	25 (21.9)	65 (16.8)
Malignant peripheral nerve sheath tumor	10 (3.7)	10 (9.0)	0	7 (6.1)	17 (4.4)
Leiomyosarcoma	22 (8.0)	11 (9.9)	11 (6.8)	11 (9.7)	33 (8.5)
Undifferentiated pleomorphic sarcoma	67 (24.5)	28 (25.2)	39 (23.9)	40 (35.1)	107 (27.6)
Myxofibrosarcoma^a^	37 (13.5)	0	37 (22.7)	0	37 (9.5)
Unclassified spindle cell^a^	8 (2.9)	1 (0.9)	7 (4.3)	0	8 (2.1)
Pleomorphic liposarcoma^a^	14 (5.1)	1 (0.9)	13 (8.0)	0	14 (3.6)
Pleomorphic rhabdomyosarcoma	6 (2.2)	0	6 (3.7)	0	6 (1.6)
Radiotherapy					
Preoperative	117 (42.7)	18 (16.2)	99 (60.7)	17 (14.9)	134 (34.5)
Postoperative	122 (44.5)	76 (68.5)	46 (28.2)	81 (71.1)	203 (52.3)
Preoperative and postoperative	9 (3.3)	2 (1.8)	7 (4.3)	1 (0.9)	10 (2.6)
None	26 (9.5)	15 (13.5)	11 (6.8)	15 (13.2)	41 (10.6)

Abbreviation: NACT, neoadjuvant chemotherapy.

^a^ Histologies allowed only in the nonrandomized cohort of the study.

After a median (IQR) follow-up of 72 (43-93) months, 156 of 388 patients (40.2%) developed a disease recurrence. In the randomized cohort (n = 201), the median (IQR) follow-up was 86 (70-99) months, and 115 patients (57.2%) developed a disease recurrence.

**Figure 3.**
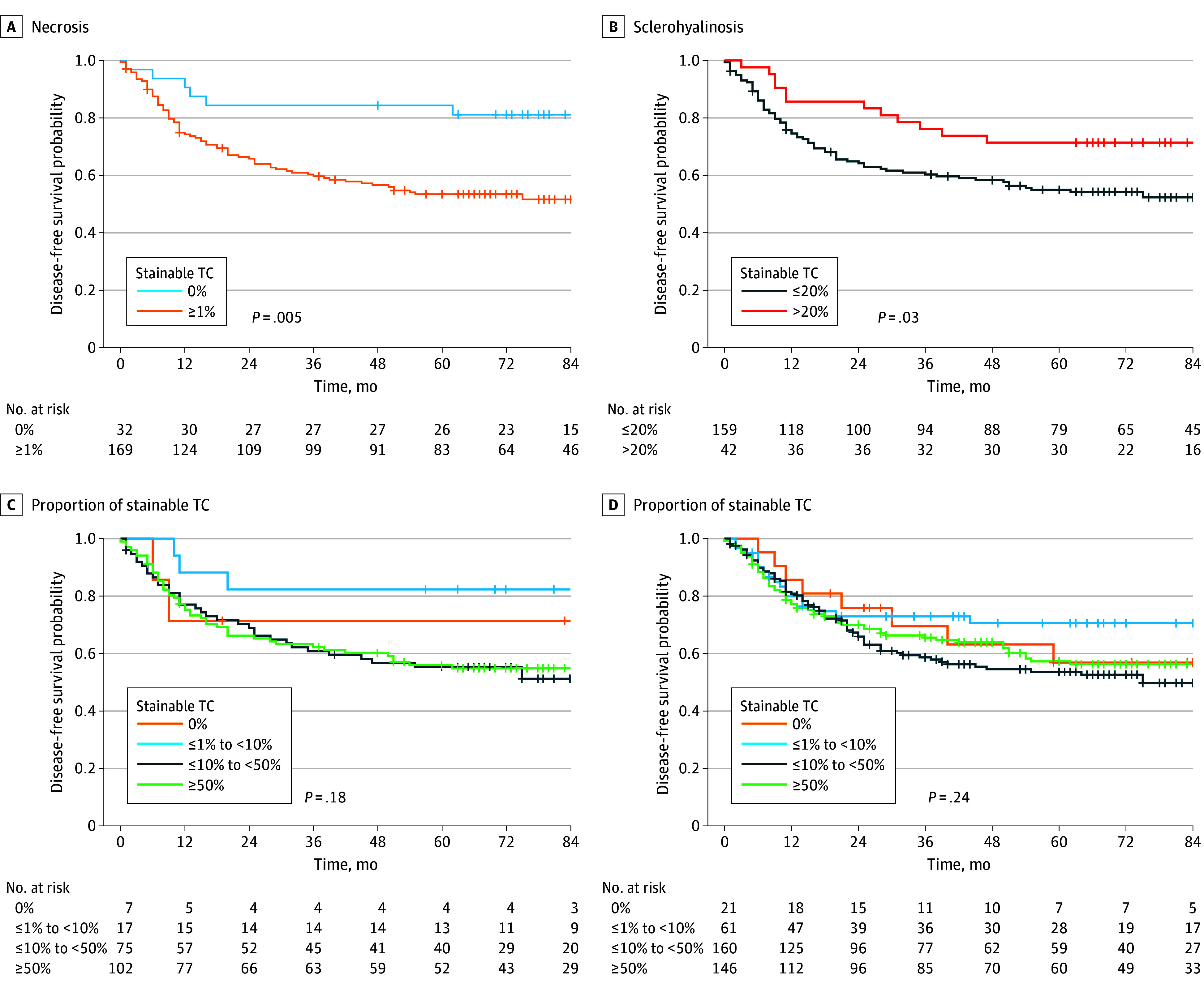
Kaplan-Meier Curves for Disease-Free Survival by Necrosis, Sclerohyalinosis, and Pathologic Tumor Response in the Randomized Cohort and by All Evaluable Patients TC indicates tumor cell.

The distribution of characteristics of histopathological response to neoadjuvant treatment suggested a relatively low prevalence for each of the considered morphological features (eTable and eFigure 2 in Supplement 2). In the randomized cohort, a proportion of 1% or more stainable tumor cells were detected in most patients (194 [96.5%]), underlying the rare occurrence of a pathological complete response in high-risk STS treated with 3 cycles of NACT. Presence (≥1%) of necrosis in 169 patients (84.1%) was associated with shorter DFS (hazard ratio [HR], 3.11; 95% CI, 1.36-7.14; *P* = .007) (Figure 3A), and greater than 20% of sclerohyalinosis in 42 patients (20.9%) was associated with a lower risk of recurrence (HR, 0.51; 95% CI, 0.28-0.94; *P* = .03) (Figure 3B). The other evaluated characteristics of histopathological response did not show any association with DFS (Table 2).

**Table 2.** Association Between Characteristics of Histopathological Response and Patient Disease-Free Survival in Patients in the Randomized Cohort of the ISG-1001

Variable and category, %	Patients, No. (%)		HR (95% CI)	*P* value
	Total (n = 201)	No association (n = 86		
Proportion of stainable tumor cells				
0, Reference group	7 (3.5)	2 (2.3)	NA	NA
>0 to <1	0	NA	NA	NA
1 to <10	17 (8.5)	3 (3.5)	0.50 (0.08-2.98)	.44
10 to <50	75 (37.3)	36 (41.9)	1.66 (0.40-6.90)	.49
≥50	102 (50.7)	45 (52.3)	1.53 (0.37-6.30)	.56
Tumor necrosis				
0, Reference group	32 (15.9)	6 (7.0)	NA	.007
≥1	169 (84.1)	80 (93.0)	3.11 (1.36-7.14)	
Hemorrhage				
0, Reference group	134 (66.7)	56 (65.1)	NA	.44
≥1	67 (33.3)	30 (34.9)	1.19 (0.76-1.86)	
Fibrohistiocytic reaction with hemosiderin				
0, Reference group	165 (82.1)	69 (80.2)	NA	.28
≥1	36 (17.9)	17 (19.8)	1.34 (0.79-2.28)	
Fibrosis or sclerosis				
0, Reference group	140 (69.7)	53 (61.6)	NA	.05
≥1	61 (30.3)	33 (38.4)	1.54 (1.00-2.38)	
Sclerohyalinosis^a^				
≤20, Reference group	159 (79.1)	74 (86.0)	NA	.03
>20	42 (20.9)	12 (14.0)	0.51 (0.28-0.94)	

Abbreviations: HR, hazard ratio; NA, not applicable.

^a^ Sclerohyalinosis was categorized on the second tertile.

The proportion of stainable tumor cells was not associated with DFS either when dichotomized as present or absent (HR, 1.47; 95% CI, 0.36-5.98; *P* = .59) or when categorized according to the EORTC-STBSG classification (Table 2, Figure 3C). Only 7 patients (3.5%) had absence of stainable tumor cells or a proportion of stainable tumor cells lower than 1%. To further investigate these findings, patients who had neoadjuvant RT together with NACT (32 [15.9%]) were excluded from this analysis, leaving patients treated only with NACT. The findings confirmed that the presence of necrosis in 142 (84.1%) was associated with shorter DFS (HR, 3.06; 95% CI, 1.23-7.60; *P* = .02), while greater than 20% of sclerohyalinosis detected in 31 patients (18.3%) was associated with a reduced risk of recurrence (HR, 0.45; 95% CI, 0.21-0.93; *P* = .03). The main outcome of the ISG-STS 1001 trial suggested that anthracycline plus ifosfamide was the regimen preferred for high-risk patients compared with histotype-tailored NACT. Consequently, we considered only patients treated with anthracycline plus ifosfamide in the randomized cohort and observed the association between presence of sclerohyalinosis higher than 20% and lower risk of recurrence (24 of 98 [24.5%]; HR, 0.24 [95% CI, 0.09-0.67], *P* = .007). Tumor necrosis remained associated with shorter DFS in patients who received histotype-tailored NACT (84 of 103 [85.4%]; HR, 9.78 [95% CI, 1.35-71.18], *P* = .02), supporting the hypothesis of necrosis being tumor-intrinsic rather treatment-related.

To increase the robustness of these results, both cohorts of randomized and nonrandomized patients were analyzed together (N = 388). The analysis showed that the presence of necrosis (313 [80.6%]; HR, 2.54 [95% CI, 1.51-4.26], *P* < .01) and sclerohyalinosis greater than 20% (111 [28.6%]; HR, 0.64 [95% CI, 0.44-0.93], *P* = .02) could be used to estimate DFS. The analysis of the overall patient series also confirmed the lack of association when the proportion of stainable tumor cells was dichotomized as present or absent and when categorized according to the EORTC-STBSG classification (Figure 3D). To address potential confounding from the pooled data from both randomized and nonrandomized patients, we adjusted for baseline variables and largely found consistency with the main analysis. No association between DFS and sclerohyalinosis was found.

## Discussion

To our knowledge, this secondary analysis is the first prospective study to provide an in-depth characterization of the histopathological response to NACT in patients with high-risk STS of extremities or trunk wall. The proportion of stainable tumor cells, considered as the most relevant posttreatment change in STS,^17^ did not stratify patient risk. This study supported consideration of the occurrence of more than 20% of sclerohyalinosis to identify patients with the best outcome after anthracycline-based NACT.

### Criteria for Histopathological Response

In sarcomas, criteria for stratification of histopathological response have been established for osteosarcoma^6,7^ and Ewing sarcoma.^7,18,19^ In osteosarcoma, the percentage of histological response, including necrosis, fibrosis, and calcification, was ultimately considered to be a proxy of resistance to treatment and presence of minimal residual disease.^20^ As a consequence, histological response is routinely documented in postoperative pathology report.^20^ In osteosarcoma, a response is considered good when the proportion of stainable tumor cells is 10% or less or when the histological response is 90% or greater.^6^ For Ewing sarcoma of bone, the optimal threshold is less clearly defined. Previous studies suggest that a complete (100%) response is the best indicator of a favorable outcome,^21^ whereas earlier reports classified a good response as achieving between 90% and 100% posttreatment changes.^19,22^ The same level of evidence has not been reached to date for STS, where studies have been conducted on different regimens, including systemic NACT mainly based on regimens containing an anthracycline, locoregional NACT administered through isolated limb perfusion, RT, or a combination of RT and chemotherapy.^23,24,25,26,27,28,29,30,31,32,33,34,35^ Histopathological response was retrospectively analyzed in the phase 3 clinical trial that compared 3 cycles with 5 cycles of perioperative chemotherapy.^12,23,24,36^ Histopathological posttreatment modifications after NACT correlated with tumor response detected at preoperative magnetic resonance imaging.^23^ Radiologic changes were associated with better outcomes for patients who showed a histopathological response.^24^ A substantial histopathological response (considered as a very good response) was defined as the presence of 10% or less stainable tumor cells after neoadjuvant treatments and surgery.^23^ This evidence enables the EORTC-STBSG to generate their scoring system for histopathological response given the rate of stainable tumor cells,^17^ and this scoring system has been prospectively tested in the ISG-STS 1001 study. Further analysis of the consistency between histopathological and radiologic characteristics of tumor response in a subgroup of patients warrants additional research.

In other retrospective studies, large variations exist, including but not limited to heterogeneity in STS histotypes, chemotherapy drugs and schedules, the combination of neoadjuvant RT, time interval between NACT surgery, and characteristics of histopathological response.^23,24,25,26,27,28,29,30,31,32,33,34,35^ These studies included a relatively high prevalence of patients with low-risk STS who were treated primarily with neoadjuvant RT and achieved a pathologic complete response, generally defined as the presence of only posttreatment changes and absence of stainable tumor cells in approximately 25% of patients.^26^ This result was consistent with a recent retrospective study of patients with localized STS treated with preoperative RT alone or combined with chemotherapy in the NRG Oncology/Radiation Therapy Oncology 9514 and 0630 nonrandomized clinical trials.^37^ In the present study, only a minority of patients (7 of 201 [3.5%]) had a proportion of stainable tumor cells less than 1%, and this low proportion likely explains the lack of association with patient outcomes. We cannot ascertain whether the low response rate was associated with the study design, particularly the 3 cycles of NACT, which may not lead to clinically significant histopathological response, as previously discussed.

### Schlerohyalinosis

In this study, the presence of greater than 20% of sclerohyalinosis estimated patient DFS, which is consistent with the complete substitution of tumor and tumor microenvironment with acellular sclerohyaline matrix. This feature was highlighted in a previous EORTC study of neoadjuvant RT in STS.^38^ In contrast to the proportion of stainable tumor cells, the presence of at least 20% hyalinization and fibrosis was associated with improved estimation of patient outcome and, if confirmed in future studies, could serve as a meaningful end point in neoadjuvant clinical trials. However, it remains impossible to assess the magnitude of the increased proportion of sclerohyalinosis after NACT compared with the baseline proportion.

Several STS histotypes are characterized by the transcription of gene sets related to extracellular matrix and exhibit a spectrum of proteins involved in its remodeling.^39,40^ Sclerohyalinosis was associated with outcomes in patients with melanoma treated with neoadjuvant immune checkpoint inhibitors or *BRAF/MEK-*targeted therapies for locally advanced disease.^41,42^ The association between sclerohyalinosis and DFS observed in our study suggests possible mechanisms of extracellular matrix remodeling through chemotherapy-induced changes in the tumor cells and tumor microenvironment. In the ISG-STS 1001 trial, we showed the association between anthracycline-based NACT and changes in tumor immune microenvironment, a condition that further supports the hypothesis that sclerohyalinosis is treatment-induced. In undifferentiated pleomorphic sarcoma, the NEOSARCOMICS trial also confirmed increased survival for patients with immune infiltration, although it suggested a lack of correlation between immune infiltrate and histopathological response.^43^ It remains unclear whether the identified threshold of sclerosis-hyalinosis is dependent on tumor histologic type and treatment regimen—in terms of both type of drugs used and duration of therapy—or if it can be considered as a more generalizable parameter.

### Limitations

This analysis is limited in several ways. First, it lacked validation in independent series of high-risk STS. This limitation extends to the data-driven cutoff used for sclerohyalinosis (>20%), which might restrict its generalizability and hence necessitate future studies to validate and refine this cutoff. However, surgical specimens were prospectively and systematically sampled and evaluated by pathologists with expertise in soft-tissue tumors, which resulted in an unprecedented collection of data. Second, our evaluations were limited by the inability to assess histopathological response in the tumor mass before neoadjuvant therapy. Therefore, this study cannot determine the extent to which the observed tumor characteristics after NACT are true posttreatment changes or intrinsic features of the tumor. Third, although we selected the most prevalent sarcoma histotypes in the extremities and trunk wall, it was not powered to evaluate differences in the characteristics of histopathological response for each sarcoma histotype. Therefore, we cannot rule out possible differences in response to NACT containing anthracycline plus ifosfamide with or without RT across different sarcomas, as suggested by histologic type–specific retrospective analysis in undifferentiated pleomorphic sarcoma and myxofibrosarcoma. To minimize this issue, we accounted for some differences, such as the need to adapt tumor measurements in case of cystic synovial sarcoma. The addition of neoadjuvant RT to chemotherapy may represent a substantial confounder; thus, as a mitigation strategy, we performed subgroup analyses.

Finally, it may be possible that 3 cycles of NACT may not result in a meaningful histopathological response. This study tested 3 cycles of full-dose anthracyclines plus ifosfamide, which was consistently tested following the previous randomized clinical trial performed by the Italian and Spanish sarcoma groups (ISG and GEIS) and demonstrated the noninferiority of 3 cycles over 5 cycles of anthracycline plus ifosfamide for DFS and overall survival.^36,44^ Three cycles were not meant to maximize the histopathological tumor response, which may be higher with the implementation of 5 cycles and a more extensive use of preoperative RT. Conversely, the aim of 3 cycles was to lower systemic risk and minimize toxic effect. When the intent of the preoperative treatment is local, increasing NACT and combining it with RT may be considered.

## Conclusions

The findings of this secondary analysis of a clinical trial of patients with high-risk STS treated with NACT questioned the EORTC-STBSG criteria for assessment of histopathological response to chemotherapy, making the assessment of response to radiologic imaging likely more effective at this stage.^23,24,45^ Specifically, the proportion of stainable tumor cells, considered as the most relevant posttreatment change, did not stratify patient risk and supported the consideration of presence of sclerohyalinosis (>20%) in identifying patients with the best outcome after NACT. Additionally, this study suggests caution when a clinical trial in patients with primary STS considers histopathological response as the primary end point.

## Notes

Supplement 1.
Trial Protocol

Supplement 2.
eMethods.
**eFigure 1.** Representation of Schelorohyalinosis **eFigure 2.** Distribution of the Characteristics of Histopathological Response Considered in This Study in All Evaluable Patients n=388 **eTable.** Distribution of Characteristics of Histopathological Response
eReferences

Supplement 3.
Data Sharing Statement
