# Supplementary material for: Histopathological Response After Neoadjuvant Chemotherapy for High-Risk Soft-Tissue Sarcomas: A Secondary Analysis of a Randomized Clinical Trial
Source: JAMA Netw Open. 2025 Nov 6;8(11):e2540177. doi: 10.1001/jamanetworkopen.2025.40177 (PMC12593128; doi:10.1001/jamanetworkopen.2025.40177)
Supplement: Supplement 1. — Trial Protocol [file jamanetwopen-e2540177-s001.pdf]

ALIAN SARCOMA GROUP
GRUPO ESPAÑOL de INVESTIGACIÓN de SARCOMAS
GROUPE SARCOMES FRANÇAIS
Maria Sklodowska Curie Memorial Cancer Centre
and Institute of Oncology
Warsaw, Poland

Cooperative groups for clinical and translational research into sarcomas

LOCALIZED HIGH-RISK SOFT TISSUE SARCOMAS OF THE EXTREMITIES AND TRUNK WALL IN ADULTS: AN INTEGRATING APPROACH COMPRISING STANDARD VS HISTOTYPE-TAILORED NEOADJUVANT CHEMOTHERAPY (ISG-STS 10-01)

Prospective controlled randomized trial

Amendment 3.0 12Jan2017

Study Chairman:
Alessandro Gronchi
Fondazione IRCCS Istituto Nazionale Tumori, Milano

CRO (study management and data management):
Domenico Franco Merlo
Centro Clinical Trials
IRCCS Azienda Ospedaliera Universitaria San Martino – IST
Istituto Nazionale per la Ricerca sul Cancro, Genova

Design and statistical analysis:
Paolo Bruzzi
IRCCS Azienda Ospedaliera Universitaria San Martino – IST
Istituto Nazionale per la Ricerca sul Cancro, Genova

International steering committee

1. Alessandro Gronchi (Istituto Nazionale Tumori – Milano) - Study Chair and ISG Principal Investigator
2. Javier Martin Broto (Son Espases Hospital, Palma de Mallorca) - GEIS Principal Investigator
3. Jean-Yve Blay (Centre Leon Berard - Lyon) - FSG Principal Investigator
4. Paolo Bruzzi (Istituto Tumori – Genova) – Biostatistician
5. Franco Merlo (Istituto Tumori – Genova) - Data Manager

Italian steering committee

1. Alessandro Gronchi (Istituto Nazionale Tumori – Milano)
2. Mario Mercuri (Istituto Ortopedico Rizzoli – Bologna)
3. Paolo Casali (Istituto Nazionale Tumori – Milano)
4. Piero Picci (Istituto Ortopedico Rizzoli – Bologna)
5. Stefano Ferrari (Istituto Ortopedico Rizzoli – Bologna)
6. Vittorio Quagliuolo (Istituto Clinico Humanitas – Milano)
7. Sergio Frustaci (Centro di Riferimento Oncologico – Aviano)
8. Rodolfo Capanna (Centro Traumatologico Ortopedico – Firenze)
9. Patrizia Olmi (Istituto Nazionale Tumori – Milano)
10. Angelo Paolo Dei Tos (Ospedale Generale USL9 – Treviso)
11. Silvia Stacchiotti (Istituto Nazionale Tumori – Milano)
12. Elena Palassini (Istituto Nazionale Tumori – Milano)
13. Daniel Vanel (Istituto Ortopedico Rizzoli – Bologna)
14. Carlo Morosi (Istituto Nazionale Tumori – Milano)
15. Antonella Messina (Istituto Nazionale Tumori – Milano)
16. Virginia Ferraresi (Istituto Regina Elena – Roma)
17. Gaetano Apice (Istituto Nazionale Tumori G. Pascale – Napoli)
18. Sergio Mapelli (Istituto G. Pini – Milano)
19. Vincenzo Ippolito (Oncologia Ortopedica – Brescia)
20. Tommaso Martino De Pas (Istituto Europeo di Oncologia – Milano)
21. Giovanni Grignani (Istituto Candiolo – Torino)
22. Alessandro Comandone (Ospedale Gradenigo – Torino)
23. Franco Merlo (Istituto Tumori – Genova)

24. Paolo Bruzzi (Istituto Tumori – Genova)

Spanish steering committee

1. Eduardo Ortiz (Hospital La Paz, Madrid). Surgical reviewer
2. Raquel Correa (Hospital Virgen de la Victoria, Málaga). Radiotherapy reviewer.
3. Silvia Bagué (Hospital Sant Pablo, Barcelona). Pathology reviewer.
4. Jaume Llauger (Hospital Sant Pau, Barcelona). Radiology reviewer.
5. Claudia Valverde (Hospital Vall d'Hebrón, Barcelona). Oncology reviewer
6. Javier Martin Broto (Son Espases Hospital, Palma de Mallorca) - GEIS Principal Investigator

French steering committee

1. Jean-Yve Blay (Centre Leon Berard - Lyon)

Polish steering committee

1. Piotr Rutkowski (Maria Sklodowska Curie Memorial Cancer Centre and Institute of Oncology- Warsaw)
2. Iwona Lugowska (Maria Sklodowska Curie Memorial Cancer Centre and Institute of Oncology - Warsaw)

A) AMENDAMENT RATIONALE

This major amendment stems from the results of the 3rd futility analysis carried out on May 10th 2016. Based on this analysis, the IDMC advised the SC to stop recruitment of other patients into the study. This amendment has three aims:
- to foresee the re-opening of recruitment, if any, in subgroups of patients;
- to foresee the strategy of further analyses;
- to foresee a new publication policy.

The following scientific and ethical considerations have been taken into account:

a) Based on the interim futility analysis, it is not ethically acceptable to enroll additional patients into the original study, due to the presence of a significantly worse DFS and OS in the experimental arm. However, a strong rationale (15) suggests the efficacy of the experimental therapy (trabectedin) in the Myxoid Liposarcoma subgroup. While confirming a markedly reduced toxicity of this regimen as compared to the standard, the futility analysis supports the hypothesis of an equivalent efficacy. This prompts to continue enrollment of patients of this subgroup in the randomized study, albeit with a different statistical approach (see below).
The choice to continue the enrollement in the present study instead of starting a new one, stems from 2 considerations:

I. The procedures and the organization required to start a new study would require several months to more than a year, therefore delaying the availability of the answer to this clinically relevant question for an equivalent period. Meanwhile, all new patients would continue to be treated with a potentially equivalent but more toxic therapy.
II. From a statistical viewpoint, the rationale for using trabectedine in this subgroup of patients was not generated by this subgroup analysis but was already fully available at the time the study was conceived (15). Therefore, there is no reason to consider the evidence collected from the first 64 patients enrolled in this group as hypothesis generating and to exclude it from the analysis of a hypothesis testing study.

b) Originally the final analysis was planned after the observation of 150 events. However, having stopped recruitment after 287 patients, it is unlikely that this number of events is observed in a reasonable time period, and it seems appropriate to reduce the number of events for the final analysis to 130, allowing a 80% power to detect a significant difference at the 5% 2-sided level, if the true HR is 0.6. The switch from the one-sided test used in the original statistical plan is due to the results of the futility analysis, which suggest a higher risk in the experimental arm. No further interim analysis are planned due to the closure of patient recruitment.

c) The futility analysis suggests that there is a strong benefit associated with the use of standard therapy. Since it is not apparent, nor is it conceivable, that the experimental therapy was associated with any detrimental effect, these findings provide evidence of the efficacy of neo-adjuvant therapy with full-dose anthracycline plus ifosfamide in high-risk STS. This efficacy was not uniformly found across available trials and therefore these findings may prove practice-changing for future patients. Yet evidence of efficacy from a futility analysis should be considered with caution due to issues of multiplicity (being an unplanned interim superiority analysis). Therefore, the SC and the steering committee in agreement with all investigators contributing to the study decide to publish the results of this interim analysis,

explicitly pointing to caution required in its interpretation due this statistical issue. The next publication will be based on the results of the final analysis as amended above and is expected in 2 yrs from now.

d) Randomization of patients affected by high-grade Myxoid Liposarcoma to either epirubicin + ifosfamide or Trabectedin will continue, with a non-inferiority design, in order to reject the hypothesis that trabectedin is associated with a HR of relapse = 1.25. To this aim, a Bayesian monitoring approach will be used until the probability that the true HR is higher than 1.25 will be greater than 80% or smaller than 5%. The Bayesian analysis will use as its prior the distribution of the HR computed on the first 64 patients with 8 events. This distribution will be analyzed every time 10 more events are observed. The results of this analysis will be submitted yearly to the IDMC.

e) All other patients eligible for the original study, either in the randomized or in the observational part, will be registered, treated with the standard therapy and followed accordingly to the procedures described in the protocol. The study will be closed to recruitment according to the original plan.

f) The success obtained by immunomodulatory therapy in other neoplastic diseases underlie the need to study the role of host immunity in sarcoma. This series could provide deep insights about the role of inflammatory infiltrate analyzing it both as prognostic and predictive parameter. And the presence of pretreatment biopsy and surgical specimen can also shed light on host contribution to treatment

B. CHANGES INTRODUCED IN THE PROTOCOL

3 ELIGIBILITY

3.1 Inclusion criteria according to amendment 3.0

1) Soft tissue sarcoma of adults, primary or locally recurrent, with histology, belonging to Myxoid-Round Cell Liposarcoma (cellular component >5 %) (Group 1)
   Or belonging to one of the following for the registration (Group 2):
   - Leiomyosarcoma
   - Synovial sarcoma
   - Malignant Peripheral Nerve Sheat Tumor
   - Undifferentiated Pleomorphic Sarcoma (ex Malignant fibrous histiocytoma)
   - Myxofibrosarcoma
   - Unclassified Spindle Cell Sarcoma
   - Pleomorphic Liposarcoma
   - Pleomorphic Rhabdomyosarcoma

5 STUDY DESIGN

5.1 Study description

The prospective, controlled, randomized study comparing standard with histotype-tailored chemotherapy within the context of an integrated strategy for high risk soft tissue sarcomas typical of the adult will be continued with observation of patients randomized up to May 2016, and of all patients registered after that date, that are treated with full-dose standard chemotherapy, regardless of the histologic type, with the exception of patients with Myxoid Liposarcoma (see below)

According to the results of the 3rd futility analysis carried out on May 10th 2016 it is not ethically acceptable to enroll additional patients into the original study, due to the presence of a **significantly worse DFS and OS in the experimental arm**.
However, a strong rationale (15) suggests the efficacy of the experimental therapy (trabectedin) in the Myxoid Liposarcoma subgroup. While confirming a markedly reduced toxicity of this regimen as compared to the standard, the analysis support the hypothesis of an equivalent efficacy.
As a consequence, patients with a histological diagnosis of Myxoid liposarcoma with hypercellularity (round cell MLPS) (cellular component > 5 %) will be continue randomization to receive either standard chemotherapy (Arm A) or trabectedin (Arm B2).
Radiation therapy will be also delivered in the post-operative setting as appropriate

All patients with other histotypes they will be registered and treated by standard chemotherapy as in Arm A.
Radiation therapy will be also delivered in the pre or post-operative setting as appropriate.

5.1.1 Randomized patients (Group 1)

Myxoid liposarcoma with hypercellularity (round cell MLPS) (cellular component > 5 %) will be randomized to receive standard (Arm A) versus histotype-tailored chemotherapy (Arm B2)

A: standard chemotherapy based on full-dose epirubicin + ifosfamide
B2: trabectedin

Radiation therapy will be also delivered in the post-operative setting as appropriate

Arm A foresees, in sequence:
- 3 cycles of preoperative chemotherapy with full dose epirubicin + ifosfamide;
- adequate surgery of the primary or recurrent tumor, according to the modalities described in the proper paragraph;
- radiotherapy according to the indications and modalities described in the proper paragraph.

Arm B2 forsees, in sequence:
- 3 cycles of preoperative chemotherapy with trabectedin;
- adequate surgery of the primary or recurrent tumor, according to the modalities described in the proper paragraph;
- radiotherapy according to the indications and modalities described in the proper paragraph.

5.1.2 Registered patients (Group 2)

Patients with the following histotypes:

Undifferentiated pleomorphic Sarcoma (ex MFH)
Synovial sarcoma
Malignant Peripheral Nerve Sheath Tumor (MPNST)

Leiomyosarcoma
Myxofibrosarcoma
Unclassified Spindle Cell Sarcoma
Pleomorphic Liposarcoma
Pleomorphic Rhabdomyosarcoma

will be registered into the study and will received conventional chemotherapy (Arm A)

Arm A: standard chemotherapy based on full-dose epirubicin + ifosfamide

Radiation therapy will be delivered either in the preoperative or in the post-operative setting.

5.1.3 Patients included after definitive surgery (Group 3)
Patients already undergone definitive surgery will receive the allocated treatment in the post-operative setting. Patients needing a re-excision after inadequate surgery will receive the allocated treatment before re-excision. Neither of them will be evaluable for response.

5.2 Trial design
The randomized component of this trial in its present form, revised according to the provisions of the amendments, is a **non-inferiority study**, aimed at rejecting the hypothesis that in patients with Myxoid liposarcoma with hypercellularity, trabectedin is associated with a clinically relevant detrimental effect on prognosis as compared to standard chemotherapy with full dose epirubicin + ifosfamide.

- Primary End Point:
Disease-Free Survival computed for each patient, from the date of randomization to the date of local/distant relapse or death from any cause, whichever first, or to the date of last observation while disease free.
- Secondary end-points:
    - Overall Survival (computed from the date of randomization to the date of death from any cause)
    - Objective Response Rate (assessed according to RECIST Criteria)
    - Descriptive evaluation of tumor tissue response according to dynamic MRI (both qualitative and quantitative), CEUS (quantitative) and CT scan (semiquantitative)
    - PET response in terms of % changes of both mean and maximum SUV
    - Evaluation of pathologic tumor response
    - Correlation between radiologic and pathologic patterns of tumor response

Statistical Design and Sample Size:
Based on considerations regarding the lower toxicity associated with the use of Trabectidine and the clinical relevance of DFS endpoint, the non-inferiority margin has been set at a HR of relapse = 1.25, corresponding, for example, to an absolute decrease in the projected probability of DFS at 3 years from 70% to 64%.
To this aim, a Bayesian monitoring approach will be used. The (posterior) probability that the true HR is ≥1.25 will be continuously updated, using as prior the probability distribution of the HR computed on the first 64 patients with 8 events. This distribution will be analyzed every time 10 more events are observed. The results of this analysis will be submitted yearly to the IDMC.
Randomization may continue until the (posterior) probability that the true HR is higher

than 1.25 will be greater than 80% or smaller than 5%. In the first instance, which is equivalent to stopping a study for futility, Trabectidine will be confirmed to be less effective than standard chemotherapy. In the second instance, the hypothesis of inferiority will be rejected and Trabectidine will be declared not inferior to standard chemotherapy.
As a consequence, the present trial has no fixed sample size, and, in theory, randomization might continue indefinitely (unless external circumstances occur, such as, for instance, new promising drugs become available for trial).
Considering the rate of accrual (64 ML patients) and of events (8 relapses/deaths) recorded so far during the 5 yrs duration of this trial, it can at best be projected that during the next 5 years a similar number of patients will be randomized and 20-24 events will be observed (8 among the newly randomized patients and 12-16 among the previously randomized patients). As a consequence, with a total of 28-32 events, the estimated HR will have a Confidence Interval equivalent to the point estimated multiplied/divided by 2. For instance, if the observed HR is 0.9 (favouring trabectedine) the confidence interval will be 0.45-1.8 (insufficient to reject the null hypothesis of inferiority). Inclusion in the prior of external evidence may be helpful to reduce this uncertainty, but it is clear that it will be possible to stop the trial only if extreme results are observed in favour or against trabectidine. Yet, this trial will provide much more robust evidence than presently available on which treatment decisions in these patients can be based, while allowing to stop randomization timely should it become unacceptable from an ethical viewpoint

1 INTRODUCTION

1.1 Evidence in favor of adjuvant chemotherapy

Despite optimal local treatment about 50% of soft tissue sarcoma patients eventually develop metastases and die of metastatic disease (1, 2). For this reason, over the past 4 decades several trials of adjuvant chemotherapy for adult soft tissue sarcomas were conducted. In 1997, a meta-analysis of 14 trials of adjuvant chemotherapy (3) showed that doxorubicine-based chemotherapy significantly improves time to local and distant relapse. However, the increase in overall survival was not statistically significant. A recent update of this metanalysis (4), including 4 newer randomized trials and more than 1900 patients in the survival analysis, confirmed a 10% of reduction of overall recurrence and a 6% improvement in overall survival, which reached statistical significance. This finding may be likely ascribed to the evolution in the administration of chemotherapy including higher dose of anthracyclines and the association of ifosfamide to anthracyclines. Although the superiority of combined anthracylines + ifosfamide chemotherapy has not been formally demonstrated in terms of efficacy in advanced disease (5, 6, 7), there is in fact a broad consensus on its superiority over single agent therapy in terms of antitumoral activity (5, 6). Indeed the full dose anthracycline + ifosfamide regimen was adopted in the first Italian trial (8) that provided evidence of a benefit from adjuvant chemotherapy in high risk extremity primary sarcoma. And on this basis the ISG conducted a new randomized trial without a no-therapy arm, comparing 3 and 5 courses of the full-dose combined anthracyclines + ifosfamide regimen (9).

In a different perspective, it should be considered that sarcomas are not only rare but highly heterogeneous, encompassing more than 50 distinct histotypes. In recent years, advances in biological and clinical understanding have indicated that different histotypes of soft-tissue sarcomas should be considered as distinct entities with different sensitivity to chemotherapy. Indeed the interpretation of the results of the adjuvant trials is hampered by the heterogeneity of the study populations not only in terms of different clinical presentations but also of the variety of the histotypes included. It is now recognized that distinct histotypes can be more sensitive to specific citotoxic agents. This is the case of ifosfamide in synovial sarcomas (10), taxanes in angiosarcomas (11), gemcitabine alone (12) and gemcitabine plus dacarbazine in leiomyosarcoma (13), gemcitabine and docetaxel in undifferentiated pleomorfic sarcomas (14), trabectedin in myxoid round cell liposarcomas (15), adriamycin in myxoid round cell liposarcomas (16), ifosfamide + etoposide in malignant peripheral nerve sheath tumor (ongoing SARC 006 study, unpublished data). The histotype – tailored approach has been increasingly encouraged in the advanced disease in the last years. The best example is the use of gemcitabine plus docetaxel in advanced uterine leiomyosarcoma (17).

1.2 Study rationale

Based on these considerations, there is a broad consensus in the international community that adjuvant chemotherapy should be offered to high-risk soft tissue sarcomas as an individualized option (18). Full dose anthracycline + ifosfamide is considered as a standard in STS, and was the regimen tested in several RCT's included in the meta-analysis of adjuvant chemotherapy (4). Nevertheless, it would seem logical to employ that regimen with the highest activity, in advanced disease, in that specific hystotype. Based on these considerations, the *Italian Sarcoma Group* has decided to launch a new randomized trial in patients with high-risk soft tissue sarcoma,

comparing standard full-dose anthracycline + ifosfamide vs histotype-tailored chemotherapy in five definite histotypes.

It was decided to compare 3 preoperative cycles of standard chemotherapy, identical to that studied in the 2 previous Italian trials (the results of last one of which showed equivalence between 3 and 5 cycles) with 3 preoperative cycles of a different tailored chemotherapy for each histotype (8, 9). As a consequence, in the present study only those histotypes, for which some suggestions of an elective sensitivity to a chemotherapy regimen different from full dose anthracyclines + ifosfamide do exist, will be included.

The selected histotype and the respective tailored systemic therapy are:

- Leiomyosarcoma – gemcitabine + dacarbazine (13)
- Myxoid-Round Cell liposarcoma (cellular component > 5 %) – trabectedin (15)
- Synovial sarcoma – high dose continuous infusion ifosfamide (10)
- Malignant Peripheral Nerve Sheath Tumor – ifosfamide + etoposide (SARC 006 ongoing study)
- Undifferentiated pleomorphic sarcoma (ex MFH) – gemcitabine + docetaxel (14)

These five histotypes represent roughly 85% of the cases recruited in the 2 previous italian studies on high risk extremity and trunk wall STS. They constitute **Group 1** of the study. The remaining 15% are represented by myxofibrosarcoma, unclassified spindle cell sarcoma, pleomorphic liposarcoma and pleomorphic rhabdomyosarcoma. These histotypes will also be registered and constitute **Group 2**.

This implies the need for central pathological review of the diagnosis before randomization.

This study is primarily aimed at evaluating the prognostic effect of a standard vs a tailored approach but will also serve to confirm the prognostic advantages obtained in the previous trials, considering that the study population is virtually the same as in the previous trials.

In this trial, it was decided to give chemotherapy preoperatively. This has several advantages: a) consistency with previous trials b) improvement in the surgical approach; c) assessment of both the clinical and the pathological response to standard vs tailored chemotherapy in each histotype's group, making it possible to gain information about the prognostic significance of response to chemotherapy.

According to the different clinical presentations chemotherapy will be integrated with radiotherapy, as dictated by the needs of the individual patient and the experience of the institution, to achieve the maximum probability of local control even in locally advanced cases.

Patients will be then stratified according to whether or not they will need to receive preoperative radiation therapy. Those patients whose histotype does not allow the combination of radiation therapy to their tailored treatment in the experimental arm (leiomyosarcoma, undifferentiated pleomorphic sarcoma, myxoid-round cell liposarcoma), if a preoperative RT treatment is indicated, will not be randomized. They will be then registered and treated with conventional regimen as in Group 2.

Patients not having measurable disease (either because already undergone definitive surgery or because having undergone a previously inadequate resection and therefore going to be re-excised) will also be recruited if the inclusion criteria of the study are met (**Group 3**). Patients whose histotype belongs to Group 1 will be randomized to either receive standard or histology tailored chemotherapy. Patients whose histotype belongs to Group 2 will be just registered and treated with standard chemotherapy.

It must be acknowledged that this study has low power to detect an overall difference in outcome (DFS or OS) between the entire group of patients assigned to standard chemotherapy and the entire group of patients assigned to histology-driven chemotherapy. This because it is highly unlikely that the difference in efficacy between the two arms is homogeneous across all histological subtypes, while it is possible that the experimental arm fares better than the standard in one subgroup and similarly or even worse in another.
To address this weakness an orthogonal study of response to chemotherapy as a surrogate of DFS and OS has been introduced into the trial. This study intends to extensively investigate the response to preoperative chemotherapy (both radiological and clinical and pathological) and to validate it as a surrogate endpoint by showing that it correlates with disease free survival and overall survival independently of the study arms. Should the study provide supporting (though not definitive, due to the small number involved) evidence in favor of the validity of response as a surrogate endpoint, it would be possible to predict the effects of each regimen on DFS and OS in each histologic subgroup, based on its effects on clinical or pathological response.
Patients belonging to Group 3 (randomized in the absence of measurable disease for whatever reason) will be included in the final analysis on the primary end-points of the study, though they won’t be able to participate to the study of surrogacy. They will received the whole treatment in the post-operative setting according to the arm they will have been assigned to.
On the other hand, patients belonging to Group 2 (registered and not randomized because affected by histological subtypes other than the one listed for the tailored approach), will contribute to the study of surrogacy, and not to the one of efficacy.

2 OBJECTIVES

2.1 Primary objectives

- To compare the effect on disease-free survival of full-dose standard chemotherapy with histotype-tailored chemotherapy within the context of an integrated strategy for high risk soft tissue sarcomas typical of the adult.

2.2 Secondary objectives

- To compare the effect on overall survival of full-dose standard chemotherapy vs histotype-tailored chemotherapy.
- To compare the probability of response of standard vs histotype-tailored chemotherapy in the whole population receiving primary chemotherapy.
- To determine the objective response rate, as defined by RECIST and Choi criteria induced by preoperative full-dose standard chemotherapy vs tailored chemotherapy in each different histotype.
- To determine the pathological response rate induced by preoperative full-dose standard chemotherapy vs tailored chemotherapy in each different histotype.
- To study the feasibility of integration of preoperative chemotherapy with preoperative local-regional treatments (radiotherapy).
- PET response.

2.3 Surrogacy objective

- To validate the response to preoperative chemotherapy (both radiological and pathological) as a surrogate endpoint by showing that disease free survival and

overall survival depend on response status and are independent of the treatment arm.

3 ELIGIBILITY

3.1 Inclusion criteria according to amendment 3.0

1) Soft tissue sarcoma of adults, primary or locally recurrent, with histology, belonging to Myxoid-Round Cell Liposarcoma (cellular component >5 %) (Group 1)
   Or belonging to one of the following for the registration (Group 2):
   - Leiomyosarcoma
   - Synovial sarcoma
   - Malignant Peripheral Nerve Sheat Tumor
   - Undifferentiated Pleomorphic Sarcoma (ex Malignant fibrous histiocytoma)
   - Myxofibrosarcoma
   - Unclassified Spindle Cell Sarcoma
   - Pleomorphic Liposarcoma
   - Pleomorphic Rhabdomyosarcoma

   Or belonging to either group but not being evaluable for response (re-excision after previous inadequate resection or primary definitive surgery) (Group3).

   The histological diagnosis must be made according to the WHO criteria (19) and will have to be centrally reviewed before randomization.

2) High malignancy grade: grade 3 of 3, according to Coindre (20), or grade 2 at biopsy with a radiological evidence of more than 50% of necrosis in the tumor mass.
3) Deep seated extremities, girdles and/or superficial trunk (thoracic or abdominal wall) lesion.
4) Size of primary tumor (visible or previously inadequately resected) ≥5 cm at instrumental staging (CT, MRI), or locally recurrent of any size.
5) Age ≥ 18 years.
6) ECOG performance status ≤1 (21).
7) Adequate bone marrow function:
   WBC >3.500/mm^3
   neutrophil >1.500/mm^3
   platelets >150.000/mm^3
   hemoglobin >11 g%.
8) Adequate renal (creatinine <1.3 mg%), and hepatic function (bilirubin <1.5 mg% and transaminases <2 x n.v. If ALP > 2.5 x ULN, ALP LF and/or GGT ≤ ULN).
9) Adequate cardiac function (FE ≥50%).
10) Signed informed consent.
11) Complete compliance of the participating center with the protocol requirements.

3.2 Exclusion criteria

1) Pregnancy or lactation.
2) Distant metastasis.
3) Other malignancies within past 5 years, with the exception of carcinoma *in situ* of cervix and basocellular skin cancers treated with eradicating intent.
4) Sarcoma histotypes other than those mentioned in the inclusion criteria.
5) Prior CT and/or RT.

6) Serious psychiatric disease that precludes informed consent or limits compliance.
7) Medical disease limiting survival to less than two years, limiting compliance or which in the physician's opinion might interfere significantly with the toxicity of the treatments.
8) Cardiovascular diseases resulting in a New York Heart Association Functional Status >2 (22).
9) Uncontrolled bacterial, viral or fungal infection.
10) Impossibility of ensuring adequate follow-up.
11) Failure to comply with the requirements of the present protocol leading to exclusion of the participating center.

4 STAGING

4.1 Staging procedures before study entry (Group 1-3)

1) Physical examination with measurement in cm of at least the largest tumor diameter and definition of the site, anatomical compartment, margins, mobility, consistency, relationship with vessel, nerve and bone structures, presence of satellite lymph node involvement.
2) Conventional and dynamic contrast enhanced MRI (contrast enhanced CT only if MRI is contraindicated) of local lesion with evaluation of tumor extension and necrosis.
3) Chest and upper abdomen CT scan with contrast.
4) Total body bone scintigraphy in case of clinical suspicion.
5) PET or PET/CT of the tumor (optional).
6) Double Contrast Enhanced Ultrasound of local lesion (optional).

4.2 Staging procedures after 1 cycle of CT (just before the 2nd cycle) (Group 1-2)

1) Conventional and dynamic contrast enhanced MRI (contrast enhanced CT only if MRI is contraindicated) of local lesion with evaluation of tumor extension and necrosis.
2) PET or PET/CT if performed before study entry.
3) Double Contrast Enhanced Ultrasound of local lesion if performed before study entry.

4.3 Staging procedures before surgery (Group 1-2)

1) Physical examination with measurement in cm of at least the largest tumor diameter and definition of the site, anatomical compartment, margins, mobility, consistency, relationship with vessel, nerve and bone structures, presence of satellite lymph node involvement.
2) Conventional and dynamic contrast enhanced MRI (contrast enhanced CT only if MRI is contraindicated) of local lesion with evaluation of tumor extension and necrosis.
3) Chest and upper abdomen CT scan with contrast.
4) Total body bone scintigraphy in case of clinical suspicion.
5) PET or PET/CT if performed before study entry.
6) Double Contrast Enhanced Ultrasound of local lesion if performed before study entry.

A central radiological review is foreseen.

5 STUDY DESIGN

5.1 Study description

This is a prospective, controlled, randomized study comparing full-dose standard chemotherapy with histotype-tailored chemotherapy within the context of an integrated strategy for high risk soft tissue sarcomas typical of the adult.
The prospective, controlled, randomized study comparing with histotype-tailored chemotherapy within the context of an integrated strategy for high risk soft tissue sarcomas typical of the adult wiil be continued with observation of patients randomized up to June 2016, and of all patients registered after that date, that are treated with full-dose standard chemotherapy, regardless of the histologic type, with the exception of patients with Myxoid Liposarcoma (see below)
At study entry the central pathological review will be performed in Treviso and will be an inclusion criteria to meet. In order to have a timely diagnosis the surgeon will inform the local pathologist about the eligibility of the patient before sending the material. The local pathologist will send one paraffin block to the central reviewer in parallel with the process of his diagnosis, along with work sheets, containing all useful clinical information (e.g., age, sex, tumor location, depth, size, presence of syndromes such as NF1). FISH may be performed when diagnostically relevant. Each participating center is encouraged to store one unfixed snap frozen sample for research purposes Furthermore each center is encouraged to store frozen blood sample to allow determination of germline mutation, such as NF1 or TP53 mutations.
The final reviewed diagnosis will have to be available within **2 weeks** from the biopsy date. (The case will be also identified as following: “Caso inviato per centralizzazione secondo protocollo ISG1001”).
The material will be sent to the following address: Unità Operativa Complessa di Anatomia Patologica, Ospedale Regionale Ca’ Foncello, Piazza Ospedale 1, 31100 Treviso.

As regards GEIS and GSF-GETO centers, the central pathological review will be performed respectively in Barcelona (Hospital Sant Pablo, dr. Silvia Bagué) and Bordeaux (Institut Bergonie, dr. Jean Michel Coindre). As for the Polish center, the central pathological review will be performed in Treviso.

The randomization was performed up to 26 May 2016 based on five histotype’s groups

1. Undifferentiated pleomorphic Sarcoma (ex MFH)
2. Myxoid liposarcoma with hypercellularity (round cell MLPS) (cellular component > 5 %)
3. Synovial sarcoma
4. Malignant Peripheral Nerve Sheath Tumor (MPNST)
5. Leiomyosarcoma

The following histotypes will also be included and registered, but treated only by standard chemotherapy (Group 2):

a. Myxofibrosarcoma
b. Unclassified Spindle Cell Sarcoma
c. Pleomorphic Liposarcoma
d. Pleomorphic Rhabdomyosarcoma

RANDOMIZATION PRIOR AMENDMENT 3.0

5.1.1 Randomized patients (Group 1)

Patients belonging to Group 1 were then randomized to receive standard *versus* histotype-tailored chemotherapy. The randomization was to be performed before starting the first cycle

of chemotherapy. The only stratifications was the administration of preoperative radiation therapy or not, for those histological subtypes for which the combination between the HT CT and RT is doable.
For each histotype group there were two arms of treatment:

A:
standard chemotherapy based on full-dose epirubicin + ifosfamide
B:
histotype-tailored chemotherapy which differs according to the histotype.

Five different regimens of histotype-tailored chemotherapy have been identified.
B_1: gemcitabine + docetaxel for histotype **1**
B_2: trabectedin for histotype **2**
B_3: ifosfamide for histotype **3**
B_4: ifosfamide + etoposide for histotype **4**
B_5: gemcitabine + dacarbazine for histotype **5**

Arm A foresees, in sequence:
- 3 cycles of preoperative chemotherapy with full dose epirubicin + ifosfamide;
- adequate surgery of the primary or recurrent tumor, according to the modalities described in the proper paragraph;
- radiotherapy according to the indications and modalities described in the proper paragraph.

Arm B_1 forsees, in sequence:
- 3 cycles of preoperative chemotherapy with gemcitabine + docetaxel;
- adequate surgery of the primary or recurrent tumor, according to the modalities described in the proper paragraph;
- radiotherapy according to the indications and modalities described in the proper paragraph.

Arm B_2 forsees, in sequence:
- 3 cycles of preoperative chemotherapy with trabectedin;
- adequate surgery of the primary or recurrent tumor, according to the modalities described in the proper paragraph;
- radiotherapy according to the indications and modalities described in the proper paragraph.

Arm B_3 forsees, in sequence:
- 3 cycles of preoperative chemotherapy with high dose continuous infusion ifosfamide;
- adequate surgery of the primary or recurrent tumor, according to the modalities described in the proper paragraph;
- radiotherapy according to the indications and modalities described in the proper paragraph.

Arm B_4 forsees, in sequence:
- 3 cycles of preoperative chemotherapy with ifosfamide + etoposide;
- adequate surgery of the primary or recurrent tumor, according to the modalities described in the proper paragraph;
- radiotherapy according to the indications and modalities described in the proper paragraph.

Arm B_5 forsees, in sequence:
- 3 cycles of preoperative chemotherapy with gemcitabine + dacarbazine;

- adequate surgery of the primary or recurrent tumor, according to the modalities described in the proper paragraph;
- radiotherapy according to the indications and modalities described in the proper paragraph.

Radiation therapy was delivered in the post-operative setting, unless otherwise indicated for clinical needs. If the tailored regimen was not combinable with preoperative RT, patients were excluded from group 1 and included in Group 2 (just registration and treatment by conventional chemotherapy).

5.1.2 Registered patients (Group 2)
Patients belonging to Group 2 received conventional chemotherapy as detailed for Arm A. Radiation therapy was deliverable either in the preoperative or in the post-operative setting. Patients belonging to Group 1 but needing preoperative radiotherapy, not combinable with the tailored regimen, were excluded from the randomization: they were registered and simply treated by standard CT (Arm A), as reported above.

5.1.3 Patients included after definitive surgery (Group 3)
Patients already undergone definitive surgery received all treatment in the post-operative setting. Patients needing a re-excision after inadequate surgery were allowed to receive the treatment as patients in group 1 and 2, but were not evaluable for response. If by histology belonging to group 1, they were randomized to receive either standard chemotherapy (Arm A) or histology tailored chemotherapy (Arm B). If belonging to group 2, they were registered and treated by standard chemotherapy as in Arm A. Radiation therapy was delivered in the post-operative setting as appropriate.

RANDOMIZATION ACCORDING AMENDMENT 3.0

According to the results of the 3rd futility analysis carried out on May 10th 2016 it is not ethically acceptable to enroll additional patients into the original study, due to the presence **of a significantly worse DFS and OS in the experimental arm.**
However, a strong rationale (ref) suggests the efficacy of the experimental therapy (trabectedin) in the Myxoid Liposarcoma subgroup. While confirming a markedly reduced toxicity of this regimen as compared to the standard, the analysis support the hypothesis of an equivalent efficacy.

As a consequence, patients with a histological diagnosis of Myxoid liposarcoma with hypercellularity (round cell MLPS) (cellular component > 5 %) will be randomized to receive either standard chemotherapy (Arm A) or trabectedin (Arm B2).

All patients with other histotypes they will be registered and treated by standard chemotherapy as in Arm A.
Radiation therapy will be also delivered in the post-operative setting as appropriate.

5.1.1 Randomized patients (Group 1)

Myxoid liposarcoma with hypercellularity (round cell MLPS) (cellular component > 5 %)

will be randomized to receive standard (Arm A) versus histotype-tailored chemotherapy (Arm B2)

A: standard chemotherapy based on full-dose epirubicin + ifosfamide
B2:trabectedin

Radiation therapy will be also delivered in the post-operative setting as appropriate

Arm A foresees, in sequence:

- 3 cycles of preoperative chemotherapy with full dose epirubicin + ifosfamide;
- adequate surgery of the primary or recurrent tumor, according to the modalities described in the proper paragraph;
- radiotherapy according to the indications and modalities described in the proper paragraph.

Arm B2 forsees, in sequence:

- 3 cycles of preoperative chemotherapy with trabectedin;
- adequate surgery of the primary or recurrent tumor, according to the modalities described in the proper paragraph;

radiotherapy according to the indications and modalities described in the proper paragraph

5.1.2 Registered patients (Group 2)

Patients with the following histotypes:

Undifferentiated pleomorphic Sarcoma (ex MFH)
Synovial sarcoma
Malignant Peripheral Nerve Sheath Tumor (MPNST)
Leiomyosarcoma
Myxofibrosarcoma
Unclassified Spindle Cell Sarcoma
Pleomorphic Liposarcoma
Pleomorphic Rhabdomyosarcoma

will registered into the study and will received conventional chemotherapy (Arm A)

Arm A: standard chemotherapy based on full-dose epirubicin + ifosfamide

Radiation therapy will be delivered either in the preoperative or in the post-operative setting.

5.1.3 Patients included after definitive surgery (Group 3)
Patients already undergone definitive surgery received all treatment in the post-operative setting. Patients needing a re-excision after inadequate surgery were be allowed to receive the allocated treatment before re-excision. Neither of them will not be evaluable for response.

If by histology is **<u>Myxoid liposarcoma with hypercellularity (round cell MLPS) (cellular component > 5 %)</u>** they will be randomized to receive either standard chemotherapy (Arm A) or trabectedin (Arm B2).

If belonging to other histotypes they will be registered and treated by standard chemotherapy as in Arm A.
Radiation therapy will be also delivered in the post-operative setting as appropriate.

Arm A foresees, in sequence:
- 3 cycles of preoperative chemotherapy with full dose epirubicin + ifosfamide;
- adequate surgery of the primary or recurrent tumor, according to the modalities described in the proper paragraph;
- radiotherapy according to the indications and modalities described in the proper paragraph.

Arm B_2 forsees, in sequence:
- 3 cycles of preoperative chemotherapy with trabectedin;
- adequate surgery of the primary or recurrent tumor, according to the modalities described in the proper paragraph;
- radiotherapy according to the indications and modalities described in the proper paragraph.

5.2 Trial design

This was a phase III randomized study comparing the efficacy of a neoadjuvant standard approach comprising conventional CT (epirubicin + ifosfamide) with Histotype Tailored CT The randomized component of this trial in its present form, revised according to the provisions of the amendments, is a **non-inferiority study**, aimed at rejecting the hypothesis that in patients with Myxoid liposarcoma with hypercellularity, trabectedin is associated with a clinically relevant detrimental effect on prognosis as compared to standard chemotherapy with full dose epirubicin + ifosfamide. All patients affected by all other histological subtypes will be registered and treated according to standard therapy

- Primary End Point:
  - Disease-Free Survival computed for each patient, from the date of randomization to the date of local/distant relapse or death from any cause, whichever first, or to the date of last observation while disease free.
- Secondary end-points:
  - Overall Survival (computed from the date of randomization to the date of death from any cause)
  - Objective Response Rate (assessed according to RECIST Criteria)
  - Descriptive evaluation of tumor tissue response according to dynamic MRI (both qualitative and quantitative), CEUS (quantitative) and CT scan (semiquantitative)
  - PET response in terms of % changes of both mean and maximum SUV
  - Evaluation of pathologic tumor response
  - Correlation between radiologic and pathologic patterns of tumor response

Statistical Design and Sample Size:

Sample Size: The HT approach involves substantial organizational burden and is considered clinically worthwhile if associated, overall, with a 1/3 reduction in the hazard of relapse (HR=0.667), corresponding, for instance, to a reduction in the long term risk of relapse from 40% to 27%. In the original design n order to assess such an effect with 80% power at the 5% (1-sided) significance level, 150 events (relapses or deaths) needed to be observed. It was expected that the study would have been to recruit approximately 350 patients over a 3-years period, from a pool of 500 registered patients. The final analysis would have taken place after the observation of the 150th event, which should have occurred 4-5 years after the recruitment of the 1st patient.
However, having stopped recruitment after 287 patients, it is unlikely that this number of events is observed in a reasonable time period, and it seems appropriate to reduce the number of events for the final analysis to 130, allowing a 80% power to detect a significant difference at the 5% 2-sided level, if the true HR is 0.6. The switch from the one-sided test used in the original statistical plan is due to the results of the futility analysis, which suggest a higher risk in the experimental arm. No further interim analysis are planned due to the closure of patient recruitment.
Subgroup analysis: A crucial question in this study relates to the possible different effect of HT CT, as compared to standard CT, in different histotypes. This question will be addressed in 2 ways. First, a standard subgroup analysis according to histotype will be conducted, based on the tests for histotype-by-treatment interaction and on the inspection of the appropriate Forrest plot. It is acknowledged that, due to the limited sample size and to the rarity of some of the subgroups, this subgroup analyses have very low power.
Second, should the validation study on radiological and pathological response as surrogate endpoints provide positive indications, response rate will be modeled as a binary variable and by means of a logistic regression model the interaction between treatment arm and histological subtype will be assessed. Due to the well known relationship between the effects of a treatment on the true and on a surrogate endpoint, this analysis is expected to have much more power than the one based on DFS.
As for the Myxoid liposarcoma subgroup, where randomization will continue within a non inferiority study design, based on considerations regarding the lower toxicity associated with the use of Trabectidine and the clinical relevance of DFS endpoint, the non-inferiority margin has been set at a HR of relapse = 1.25, corresponding, for example, to an absolute decrease in the projected probability of DFS at 3 years from 70% to 64%.
To this aim, a Bayesian monitoring approach will be used. The (posterior) probability that the true HR is ≥1.25 will be continuously updated, using as prior the probability distribution of the HR computed on the first 64 patients with 8 events. This distribution will be analyzed every time 10 more events are observed. The results of this analysis will be submitted yearly to the IDMC.
Randomization may continue until the (posterior) probability that the true HR is higher than 1.25 will be greater than 80% or smaller than 5%. In the first instance, which is equivalent to stopping a study for futility, Trabectidine will be confirmed to be less effective than standard chemotherapy. In the second instance, the hypothesis of inferiority will be rejected and Trabectidine will be declared not inferior to standard chemotherapy.
As a consequence, the present trial has no fixed sample size, and, in theory, randomization might continue indefinitely (unless external circumstances occur, such as, for instance, new promising drugs become available for trial).

The results of this analysis will be submitted yearly to the IDMC. Considering the rate of accrual (64 ML patients) and of events (8 relapses/deaths) recorded so far during the 5 yrs duration of this trial, it can at best be projected that during the next 5 years a similar number of patients will be randomized and 20-24 events will be observed (8 among the newly randomized patients and 12-16 among the previously randomized patients). As a consequence, with a total of 28-32 events, the estimated HR will have a Confidence Interval equivalent to the point estimated multiplied/divided by 2. For instance, if the observed HR is 0.9 (favoring trabectedine) the confidence interval will be 0.45-1.8 (insufficient to reject the null hypothesis of inferiority). Inclusion in the prior of external evidence may be helpful to reduce this uncertainty, but it is clear that it will be possible to stop the trial only if extreme results are observed in favour or against trabectidine. Yet, this trial will provide much more robust evidence than presently available on which treatment decisions in these patients can be based, while allowing to stop randomization timely should it become unacceptable from an ethical viewpoint

2. ***Validation of response as a surrogate endpoint of DFS***
A validation study will evaluate overall tumor response (both radiological and pathological) as a surrogate of DFS/OS. The present randomized trial is largely underpowered for a formal validation study according to Prentice's criteria. However, the currently available evidence indicating the validity of tumor response as a surrogate of DFS/OS in various solid tumors, provides the ideal setting for developing and applying an original approach to surrogate endpoints validation based on Bayesian concepts.

3. ***Futility analysis***
No interim analysis was planned with the aim to stop patients' accrual in the presence of positive results (demonstration of superiority of HT), because of the extremely low power of these analyses in this generally underpowered study. Conversely, yearly interim futility analyses were conducted to assess if the study hypothesis that HT was associated with a 1/3 reduction in the hazard of relapse is still viable, both in the overall randomized population and in each histological subgroups. It is useful to underline that these analyses do not affect that type I error level, and therefore do not require any correction of the significance level. These analyses will focused both on the relapse-free survival and on the response rate, and werebased on the Bayesian methodology described by Parmar (23).
Due to the small numbers involved, it wasexpected that the study will be stopped for futility only in the presence of evidence supporting inferiority (though not significantly so) of the HT as compared to standard chemotherapy in the whole study or in a specific subgroup, and as a consequence, these futility analyses wereconsidered equivalent to safety analyses. To this aim an Independent Data Monitoring Committee (IDMC) were formed from a group of experts and was reviewied the interim evaluations to ensure continued subject safety as well as tolerance and efficacy.
An earlier publication on activity of different regimens on different histotypes would have been be allowed.

As for the Myxoid liposarcoma subgroup, where randomization will continue within a non inferiority study design, a Bayesian monitoring approach will be used. The (posterior) probability that the true HR is ≥1.25 will be continuously updated, using as prior the probability distribution of the HR computed on the first 64 patients with 8 events. This distribution will be analyzed every time 10 more events are observed. The results of this analysis will be submitted yearly to the IDMC.

6. CRITERIA FOR REMOVING PATIENTS FROM THE STUDY

- Appearance of metastases or progression as defined by RECIST during treatment.
- Intercurrent disease or toxicities or adverse events requiring discontinuation of treatment.
- Refusal of the patient, at any time and for any reason, to continue treatment within the study. In this case, the patient will receive the best treatment according to his/her wishes.

7 TREATMENT PLAN

7.1 Standard chemotherapy

Central venous catheter is recommended.

Arm A: epirubicin + ifosfamide

Arm A foresees 3 cycles of preoperative chemotherapy, each cycle will be repeated every 21 days and includes:

- epirubicin 60 mg/m2/day, short infusion, days 1 and 2
- ifosfamide 3 g/m2/day, days 1, 2, 3
- mesna 1000 mg/m2 x 3/day (every 3-4 h), days 1, 2, 3
- hydration with >2000 cc/day, days 1, 2, 3
- adequate antiemetic cover with anti–HT3 and steroids.

In the interval period, prophylactic use of G-CSF (filgrastim or peg-filgrastim) is recommended. Serial complete blood counts must be performed at the following times: from day +9 at least three times, possibly on alternate days, up to hematological recovery.

Dose reductions

On Day 22 treatment will be postponed one week in the event of:

- neutrophils <1000/mmc
- platelets <100.000/mmc
- non-hematological toxicities of grade 2 or higher.

If these conditions still persist on Day 29, the patient will be withdrawn from the study program and will be treated at the discretion of the physician responsible, unless only neutropenia persists with neutrophils >1000/mmc and/or thrombocytopenia with platelets >100.000/mmc, in which case the cycle will be performed at 66% of the dose of epirubicin and ifosfamide.

The chemotherapy doses of each cycle after the first will be modified on the basis of the toxicities induced by the preceding cycle according to the following scheme. The level 1 reduction will be applied if the preceding cycle was carried out at 100% of the drug dose. The level 2 reduction will be applied if the preceding cycle was carried out at 75% of the drug dose. The level 3 reduction, which will be applied if the preceding cycle was carried out at 66% of the dose, involves removal of the patient from the study and treatment at the discretion of the physician responsible.

REDUCTION LEVELS	PARAMETER	VALUES	EPI	IFX
Level 1	ANC<br>**or** PLT<br>or non hematologic toxicity	≤500/mmc for ≥5 days<br>≤25.000/mmc<br>G3-G4	75%	75%
Level 2	ANC<br>**or** PLT<br>or non hematologic toxicity	≤500/mmc for ≥5 days<br>≤25.000/mmc<br>G3-G4	66%	66%
Level 3	ANC<br>**or** PLT<br>or non hematologic toxicity	≤500/mmc for ≥5 days<br>≤25.000/mmc<br>G3-G4	**WITHDRAW FROM TRIAL**	

Anemia does not require a dosage reduction.
If lower grade toxicities are seen at the subsequent cycle the possibility of returning to the next highest dose level will be evaluated.
Chemotherapy will always be administered for the duration foreseen by the protocol (epirubicin in 2 days; ifosfamide in 3 days).

7.2 Histotype-tailored chemotherapy

Central venous catheter is recommended

Arm B_1: gemcitabine + docetaxel

Arm B_1 foresees 3 cycles of preoperative chemotherapy, each cycle will be repeated every 21 days and includes:

- gemcitabine: 900 mg/m2 on days 1 and 8 intravenously over 90 min
- docetaxel: 75 mg/m2 on day 8 intravenously over 1 h
- adequate antiemetic cover with anti–HT3 and steroids
- pre-medication with dexamethasone 8 mg (or equivalent dose prednisone) orally for 2 doses the day prior to docetaxel, and 8 mg orally twice daily for the next day
- early intervention with diuretics is encouraged for signs of docetaxel related fluid retention.

In the interval period, prophylactic use of G-CSF (filgrastim or peg-filgrastim) is recommended.

Dose reductions

On Day 8 treatment will be postponed one week in the event of:

- neutrophils <1000/mmc
- platelets <100.000/mmc
- non-hematological toxicities of grade 2 or higher

If these conditions still persist on Day 15, the patient will be withdrawn from the study program and will be treated at the discretion of the physician responsible, unless only neutropenia persists with neutrophils >1000/mmc and/or thrombocytopenia with platelets >100.000/mmc, in which case the cycle will be performed at 66% of the dose of gemcitabine and docetaxel.

On Day 22 (Day 1, second or third cycle) treatment will be postponed one week in the event of:

- neutrophils <1000/mmc
- platelets <100.000/mmc

- non-hematological toxicities of grade 2 or higher

If these conditions still persist on Day 29, the patient will be withdrawn from the study program and will be treated at the discretion of the physician responsible, unless only neutropenia persists with neutrophils >1000/mmc and/or thrombocytopenia with platelets >100.000/mmc, in which case the cycle will be performed at 66% of the dose of gemcitabine and docetaxel.

The chemotherapy doses of each cycle after the first will be modified on the basis of the toxicities induced by the preceding cycle according to the following scheme. The level 1 reduction will be applied if the preceding cycle was carried out at 100% of the drug dose. The level 2 reduction will be applied if the preceding cycle was carried out at 75% of the drug dose. The level 3 reduction, which will be applied if the preceding cycle was carried out at 66% of the dose, involves removal of the patient from the study and treatment at the discretion of the physician responsible.

REDUCTION LEVELS	PARAMETER	VALUES	GEM	Docetaxel
Level 1	ANC<br>**or** PLT<br>or non hematologic toxicity	≤500/mmc for ≥5 days<br>≤25.000/mmc<br>G3-G4	75%	75%
Level 2	ANC<br>**or** PLT<br>or non hematologic toxicity	≤500/mmc for ≥5 days<br>≤25.000/mmc<br>G3-G4	66%	66%
Level 3	ANC<br>**or** PLT<br>or non hematologic toxicity	≤500/mmc for ≥5 days<br>≤25.000/mmc<br>G3-G4	**WITHDRAW FROM TRIAL**	

Arm B_2: trabectedin

Arm B_2 foresees 3 cycles of preoperative chemotherapy, to be repeated every 21 days with trabectedin as follows:

- trabectedin: 1.3 mg/m2, given in 24-hour continuous infusion
- adequate antiemetic cover with anti–HT3 and steroids
- pre-medication with dexamethasone 4 mg per os bid the day before starting chemotherapy and for the next 2 days after having completed trabectedin infusion
- anticoagulant therapy with low molecular weight heparin (the day before starting chemotherapy and for the next eight days) for prophylaxis of central line thrombosis is optional and recommended in presence of risk factors for thrombosis

On Day 22 treatment will be postponed one week in the event of:

- neutrophils <1500/mmc
- platelets <100.000/mmc
- bilirubin >ULN
- transaminases >2.5 x ULN
- creatine phosphokinase (CPK) >2.5 x ULN
- alkaline phosphatase >2.5 x ULN (consider hepatic isoenzymes 5-nucleotidase or GGT, if the elevation could be osseous in origin)
- haemoglobin < 9 g/dl
- any other grade 3 or 4 adverse reactions (such as nausea, vomiting, fatigue)

If these conditions still persist on Day 29, the patient will be withdrawn from the study program and will be treated at the discretion of the physician responsible.

Additional monitoring of haematological parameters bilirubin, alkaline phosphatase, aminotransferases and CPK should occur weekly during the first two cycles of therapy, and at least once between treatments in subsequent cycles.

Dose reductions

Prior to re-treatment, patients must fulfill the baseline criteria defined above.

The chemotherapy doses of each cycle after the first will be modified on the basis of the toxicities induced by the preceding cycle according to the following scheme. The level 1 dose will be applied if the preceding cycle was carried out at 100% of the drug dose. The level 2 dose will be applied if the preceding cycle was carried out according to level 1 drug dose. The level 3 reduction, which will be applied if the preceding cycle was carried out according to level 2 drug dose, involves removal of the patient from the study and treatment at the discretion of the physician responsible.

REDUCTION LEVELS	PARAMETER	VALUES	Trabectedin
Level 1	ANC<br><br>**or** PLT<br>**or** bilirubin<br>**or** AST/ALT<br>**or** ALP<br>**or** any other toxicity	≤500/mmc for ≥5 days or associated with fever or infection<br>≤25.000/mmc<br>>ULN<br>≥G3 not recovered <G1<br>>2.5 x ULN<br>G3-G4	1.1 mg/m2
Level 2	ANC<br><br>**or** PLT<br>**or** bilirubin<br>**or** AST/ALT<br>**or** ALP<br>**or** any other toxicity	≤500/mmc for ≥5 days or associated with fever or infection<br>≤25.000/mmc<br>> ULN<br>≥G3 not recovered <G1<br>>2.5 x ULN<br>G3-G4	0.9 mg/m2
Level 3	ANC<br><br>**or** PLT<br>**or** bilirubin<br>**or** AST/ALT<br>**or** ALP<br>**or** any other toxicity	≤500/mmc for ≥5 days or associated with fever or infection<br>≤25.000/mmc<br>> ULN<br>≥G3 not recovered <G1<br>>2.5 x ULN<br>G3-G4	**WITHDRAW FROM TRIAL**

Anemia does not require a dosage reduction.

Once a dose has been reduced because of toxicity, dose escalation in the subsequent cycles is not recommended.

Arm B_3: high dose continuous infusion ifosfamide

Arm B_3 foresees 3 cycles of preoperative chemotherapy with high-dose ifosfamide in monotherapy. Each cycle of high-dose ifosfamide will consist of ifosfamide 14 g/m2, given in 14 days by means of an external infusion pump, every 28 days. Infusion pumps allow a 7-day continuous infusion. Therefore, the first portable pump filled with ifosfamide 7 g/m2, plus mesna 7 g/m2, will be substituted after 7 days with another.

No dose reductions are allowed. On day 8, treatment will be interrupted in case of

- platelets <100,000/mm3
- neutrophil <1,000/mm3
- creatinine > ULN

On day 29, treatment will be postponed until recovery in case of:
- platelets <100,000/mm3
- neutrophil <1,000/mm3
- creatinine > ULN

If recovery does not occur within 5 weeks (35 days) after the day 1 of last cycle the patient will be withdrawn from the study program.
In case of non hematological toxicities of grade 3 or higher treatment withdrawal will be considered.

Arm B_4: ifosfamide + etoposide
Arm B_4 consists in 3 preoperative cycles, each cycle will be repeated every 21 days and includes:
- etoposide 150 mg/m2/day, days 1, 2, 3
- ifosfamide 3g/m2/day, days 1, 2, 3
- mesna 1000 mg/m2 x 3/day (every 3-4 h), days 1, 2, 3
- hydration with >2000 cc/day, days 1, 2, 3
- adequate antiemetic cover with anti–HT3 and steroids.

In the interval period, prophylactic use of G-CSF (filgrastim or peg-filgrastim) is recommended.

Dose reductions
On Day 22 treatment will be postponed one week in the event of:
- neutrophils<1000/mmc
- platelets <100.000/mmc
- non-hematological toxicities of grade 2 or higher

If these conditions still persist on Day 29, the patient will be withdrawn from the study program and will be treated at the discretion of the physician responsible, unless only neutropenia persists with neutrophils >1000/mmc and/or thrombocytopenia with platelets >100.000/mmc, in which case the cycle will be performed at 66% of the dose of etoposide and ifosfamide.

The chemotherapy doses of each cycle after the first will be modified on the basis of the toxicities (neutrophil and platelet nadirs) induced by the preceding cycle according to the following scheme. The level 1 reduction will be applied if the preceding cycle was carried out at 100% of the drug dose. The level 2 reduction will be applied if the preceding cycle was carried out at 75% of the drug dose. The level 3 reduction, which will be applied if the preceding cycle was carried out at 66% of the dose, involves removal of the patient from the study and treatment at the discretion of the physician responsible.

REDUCTION LEVELS	PARAMETER	VALUES	IFX	VP-16
Level 1	ANC<br>**or** PLT<br>or non hematologic toxicity	$\le$500/mmc for $\ge$5 days<br>$\le$25.000/mmc<br>G3-G4	75%	75%
Level 2	ANC	$\le$500/mmc for $\ge$5 days	66%	

	**or** PLT<br>or non hematologic toxicity	≤25.000/mmc<br>G3-G4		66%
Level 3	ANC<br>**or** PLT<br>or non hematologic toxicity	≤500/mmc for ≥5 days<br>≤25.000/mmc<br>G3-G4	**WITHDRAW FROM TRIAL**	

Anemia does not require a dosage reduction.
If lower grade toxicities are seen at the subsequent cycle the possibility of returning to the next highest dose level will be evaluated.

Arm B_5: gemcitabine + dacarbazine
Arm B_5 consists in 3 preoperative cycles, each cycle will be repeated every 14 days and includes:

- gemcitabine: 1800 mg/m2 on day 1 intravenously over 180 min
- dacarbazine: 500 mg/m2 on day 1 intravenously over 20 min
- adequate antiemetic cover with anti–HT3 and steroids

In the interval period, prophylactic use of G-CSF (filgrastim or peg-filgrastim) is recommended.

Dose reductions
On Day 15 treatment will be postponed one week in the event of:

- neutrophils <1000/mmc
- platelets <100.000/mmc
- non-hematological toxicities of grade 2 or higher

If these conditions still persist on Day 22, the patient will be withdrawn from the study program and will be treated at the discretion of the physician responsible, unless only neutropenia persists with neutrophils >1000/mmc and/or thrombocytopenia with platelets >100.000/mmc, in which case the cycle will be performed at 66% of the dose of gemcitabine and dacarbazine.

The chemotherapy doses of each cycle after the first will be modified on the basis of the toxicities (neutrophil and platelet nadirs) induced by the preceding cycle according to the following scheme. The level 1 reduction will be applied if the preceding cycle was carried out at 100% of the drug dose. The level 2 reduction will be applied if the preceding cycle was carried out at 75% of the drug dose. The level 3 reduction, which will be applied if the preceding cycle was carried out at 66% of the dose, involves removal of the patient from the study and treatment at the discretion of the physician responsible.

REDUCTION LEVELS	PARAMETER	VALUES	GEM	DTIC
Level 1	ANC<br>**or** PLT<br>or non hematologic toxicity	≤500/mmc for ≥5 days<br>≤25.000/mmc<br>G3-G4	75%	75%
Level 2	ANC<br>**or** PLT<br>or non hematologic toxicity	≤500/mmc for ≥5 days<br>≤25.000/mmc<br>G3-G4	66%	66%
Level 3	ANC	≤500/mmc for ≥5 days	**WITHDRAW**	

	**or** PLT<br>or non hematologic toxicity	<25.000/mmc<br>G3-G4	**FROM TRIAL**

Anemia does not require a dosage reduction.
If lower grade toxicities are seen at the subsequent cycle the possibility of returning to the next highest dose level will be evaluated.

7.3 Supporting therapy

Oral antibiotic therapy will be given from the first cycle with:
Large spectrum antibiotics PO (unless not tolerated by the patient or at the physician's discretion).
Febrile neutropenia lasting >3 days will be treated as a general rule with broad spectrum antibiotics given intravenously with the patient hospitalized.

8. SURGERY

Surgical resection must be performed by a surgeon belonging to a center with recognized experience in treating sarcoma patients.

Biopsy must be performed at the end of the pre-operative staging and must be open incisional or large core needle biopsy (Tru-cut). Since a parallel central review of histological diagnosis is requested by the study, both the local and the reference pathologist must receive an adequate amount of tissue to express a definite diagnosis on the histologic type (only some histologic types are included in the trial) and histologic grade (only high grade sarcomas are included in the trial). Should the Tru-cut biopsy fail to provide adequate material for a definite diagnosis, incisional biopsy is mandatory.
If translational studies are to be considered one or more fresh frozen samples should be taken and stored.
Since preoperative chemotherapy and radiotherapy are foreseen in this study, the diagnosis on the surgical specimen will certainly be altered by the treatment already performed: details of histologic type may no longer be identifiable and it may be impossible to determine the grade reliably. In planning the surgical approach for biopsy it must be borne in mind that:

- The scar and the biopsy track must be included en bloc in the subsequent surgical procedure (this also applies to needle biopsy);
- The incision must always be longitudinal to the limb in the case of sarcoma of the extremities, whereas is must follow the direction of the subsequent demolition in the case of sarcoma of the superficial trunk;
- The biopsy track must always go directly to the tumor, through the muscle fibers with minimum use of retractors.;
- The biopsy track must contaminate only the anatomical compartment in which the tumor is situated;
- Very careful hemostasis must be ensured, to avoid post-surgical hematoma. The drain – if needed – should be put in line with the scar.

Surgical resection must be performed according to the following criteria. The incision must always be made along the major axis of the tumor-bearing anatomical compartment and must include the biopsy track en bloc. The quality of the surgery will be defined by its worst margin and will be classically classified as follows:

- Intralesional: when macroscopic tumor residue is left in situ.
- Marginal: when the tumor surface emerges macroscopically at the resection surface, or when microscopic tumor extension is present at the margin of resection, but without evidence of macroscopic residual disease. It will be important for the pathologist to examine the specimen with the surgeon so that correct orientation is ensured for accurate evaluation of the margins. The surgeon must help the pathologist to identify the most critical resection margin and likewise must ensure that points where the tumor emerges only due to muscle retraction following surgical removal are not identified as critical margins.
- Wide: when the tumor is covered at every point by healthy tissue (the width of the margin varies according to the type of tissue: 1 cm if muscle or fatty tissue, 1 mm. or less, if anatomical barrier (such as muscle fascia, adventitia, epineurium, periostium).
- Radical: when the tumor is removed en bloc with the entire muscular compartment and covered by intact deep fascia. If infiltration of the deep fascia is detected, surgery must be extended to include demolition of the structures involved (adjacent muscle compartment, bone, blood vessel or nerve). For tumors with an extracompartmental origin the only procedure considered radical will be amputation.
- Contaminated: when accidental rupture of the tumor pseudocapsule with spillage of material into the operating field occurs, and also when the pseudocapsule has simply emerged at the margin of resection. In these cases spillage of material must be controlled by all means, and then the operating field must be rapidly washing and the resection margins, if possible, widened. The contamination must be reported in the description of the surgical procedure and will be followed by complementary radiotherapy.

Only wide or radical resections are defined as adequate. Marginal resections in sites where wider resection is not possible may also be acceptable, provided they are always followed or preceded by radiotherapy. In particular, irrespective of the site: surgery will be largely planned on the basis of imaging findings (CT, MRI) and the least favorable intraoperative situations will be hypothesized; a wide cutaneous incision will be made along traditional lines, including en bloc the scar and the holes of previous biopsies or surgery. Once the skin-fat flaps have been prepared the tumor will be isolated within the tumor-bearing structure, with prompt recognition and careful dissection of the main vascular structures and motor nerves (femoral, sciatic, sciatic-popliteal, external/internal, median, ulnar and radial). These structures must not show tumor infiltration. Care must be taken to avoid contamination of the surgical field, which can also occur if the tumor is allowed to emerge on the surface of resection. In any case surgery in which minimal contamination has occurred will be considered acceptable, but complementary radiation therapy will have to be planned in any case. Once the malignancy has been isolated, it must be removed en bloc with the surrounding soft tissue, covered at every point by at least one centimeter of healthy tissue or less if constituted by an anatomical barrier (see above). Compartmental operations will be performed only if made necessary by the site and dimensions of the tumor. If the lesion is near structures such as the vascular-nervous fascia or bone, it must be cautiously prepared by also removing the fascia covering said structures (muscle fascia, vascular adventitia, epineurium or periostium). If these barriers are also found to be infiltrated, the underlying structures should be resected en bloc with the tumor, assessing the possibility of performing vascular, neurological or bone reconstruction as an alternative to demolitive procedures.

If surgery has been performed in centers without recognized experience in treating sarcoma patients and has been judged inadequate, re-resection must be performed. Before the patient is recruitable the original histological slides need to be centrally reviewed. The principles of surgical technique will be those described above for removal of the mass, taking care to remove the entire scar of the previous surgery en bloc, following cautious dissection of the vascular-nervous fascia and preparation of the bone if necessary.

9. RADIATION THERAPY

All patients who are candidates for conservative surgery, including re-excision of the surgical bed after surgery performed elsewhere and judged inadequate, should be considered for radiation therapy. The modalities of integration with chemotherapy will be described in the specific subparagraphs concerning the pre- and postoperative settings. Concomitant administration of radiation therapy and chemotherapy will be allowed, as well as the sequential modality.

Post-operative external beam radiation therapy alone or a combination of perioperative brachytherapy or IORT and external beam radiation therapy, must be performed in all patients undergoing conservative surgical resection according to ESMO or SIOT guidelines.

All patients in whom conservative resection with wide margins is not foreseeable at diagnosis for sites of occurrence in close proximity to known critical structures such as bone, vessels and nerves, shall preferably undergo preoperative radiation therapy.

9.1. Postoperative radiation therapy.

Postoperative radiotherapy is delivered in:

- Malignant tumors > 5cm;
- High grade and deep tumors;
- Marginal resection (microscopic: tumor close to bone, vessel or nerves or macroscopic tumor residual)
- Post radicalization in case of inadequate primary surgery

Postoperative radiation therapy will begin within 90 days after surgery.
If it’s requested it will be possible a radiation treatment concomitant to chemotherapy including Epi/Ifo; HD Ifo; Eto/ifo. Otherwise it will be postponed at the end of the chemotherapy treatment for the other regimens.

Postoperative Radiotherapy technique
A CT study must be performed to define volume of interest (VOI) and valuate the dose distribution. The standard treatment is based on 3D conformal radiotherapy; in case of the target shape would be complex or large it will be possible planning the treatment with intensity modulated radiation therapy (IMRT).

- **Target volume**: The initial target volume (**CTV2**), identified by CT and/or MRI, must include the tumor bed (referring to preoperative imaging) and the surgical bed, including the scar and the drainage site (if this does not coincide with the main incision) with craniocaudal margins of 3 cm and radial margins of 1 cm (Planned Target Volume: **PTV2**).

- **Boost:** Tumor bed (**CTV1**) with craniocaudal margin of 1-2 cm and radial margin of 0.5 cm (**PTV1**)
- **Energy**: High-energy photons between 6 -15 MV will be used.
- **Dose and fractioning**: The total planned dose is 60-66Gy (44-50 Gy + 10-16 Gy boost) on two sequential target volumes identified as described below, delivered with conventional fractioning of 2 Gy/day, 5 days a week.

In case of R1 or R2, the boost dose delivered will be 20-24 Gy.
In case of IMRT technique it's possible a simultaneous integrated boost (SIB) for a total dose in PTV1 of 56-60 Gy.

The deviation in the distribution of the isodoses should preferably be in accordance with ICRU 62. Computerized dosimetric evaluation combined with the use of customized immobilizers will allow the most suitable radiation techniques to be used for optimization of the treatment. The isocenter of treatment's fields must be checked with orthogonal radiographs at the beginning of the radiation-therapy and during the treatment, weekly orthogonal portal imaging must be acquired to control the repeatability of treatment and to reduce set-up errors.
The accuracy parameters for the treatment planning are:
$V95_{PTV2}$ (the volume included in isodose 95% of the prescribing dose) ≥ 95%
$V95_{PTV1}$ ≥ 95%.

9.2. Brachytherapy
BT will be performed alone, with postoperative ERT (anticipated boost), with preoperative ERT (postoperative boost) or in case of recurrence not subject to surgery or ERT.
Brachytherapy (BT), in centers where this procedure is available, is a valid technique that allows high dose delivery reducing normal tissue exposition.
The association with external radiotherapy (ERT) plays an important role in case of R1/R2 status and radicalization can't performed. In this way it will be possible to reduce neoplastic regeneration killing residual tumor microfoci and spare neurovascular structures close to tumor bed.

Technique
BT will be performed using after loading catheters intraoperatively on the tumor bed. The target volume will be evaluated simultaneously by the surgeon and the radiation therapist and will include the tumor bed with a margin of at least 2 cm in radial direction and 3-5 cm in longitudinal direction. The catheters, inserted percutaneously at least 2 cm from the incision and perpendicular thereto, can be fixed with absorbable suture to the deep planes to better ensure the geometry of the implant. They will be fixed with special clips at the skin exit site. Within one week of surgery, or more depending upon the course of wound healing in the postoperative period, the implant will be loaded with the 192 Ir wires, after orthogonal radiographs or CT images have been obtained for the three-dimensional reconstruction and computerized dosimetric calculation. A dose between 15-25 Gy will be delivered.
Two techniques are advisable:
- LDR (low dose rate) low radiation activity (10-15 Gy delivered/24h). The dose delivered will be 15-25 Gy + 45 Gy (ERT). Hospitalization is necessary.
- HDR (high dose rate) high radiation activity (1Gy delivered/1 minute). The dose delivered 15-21 Gy + 45 Gy (ERT). The BT procedure will be out-patient.

External beam radiation therapy will be started 3-4 weeks from the end of the brachytherapy, after completion of the first cycle of postoperative chemotherapy, if foreseen, with the same modalities as for radiation therapy alone in its first widest volume part up to the total dose of 45 Gy.

IORT

The anticipated boost can also be given with intraoperative radiotherapy (IORT) in centers where this procedure is available. The planned dose is 10-12 Gy, for negative or microscopic margins defined at surgery, prescribed at the isodose of 90% using electron beams. The energy of the electrons used must include the target volume, in the 90% isodose. Use of more than one field is allowed, taking care to avoid overlapping. The planned dose limit to critical structures (peripheral nerves) is 12 Gy; the extension of surgical margins will be defined at surgery.

9.3. Preoperative radiation therapy

Preoperative radiotherapy is recommended in:

- Locally advanced sarcoma, to create a pseudo-capsule and improve the wide surgery;
- Locally advanced sarcoma close to vessels, nerves or bone (planned free miocutaneous flap, to reduce healing complications related to post-radiation fibrosis)

Preoperative radiation therapy will after the first cycle of neoadjuvant chemotherapy and possibly not after the second in case of standard regimens.

Radiotherapy technique

- **Energy**. As in paragraph above.
- **Dose and fractioning**. The total planned dose is 44 – 50 Gy delivered with conventional fractioning of 2 Gy/day for 5 days a week.
- **Target volume.** The target volume, identified by CT and/or MRI, must include the primary lesion (Gross Tumor Volume, GTV), including the area of perilesional edema and the incisional biopsy wound, referring to the diagnostic imaging (Clinical Target Volume, CTV) with craniocaudal margins of 3 cm and radial margins of 1.5 cm for PTV (Planned Target Volume). The isodose distribution within the target volume must be evaluated on the CT study considering these parameter:
  $V95_{PTV}$ (the volume included in isodose 95% of the dose prescription) ≥ 95% or
  $V90_{PTV}$ > 95%
  The use of customized immobilizing devices will allow the most suitable radiation techniques to be used for optimization and reproducibility of the treatment. It must check the isocenter of treatment's fields with orthogonal radiographs at the beginning of the radiation-therapy and during the treatment orthogonal portal imaging must be acquired to control the accuracy and to reduce set-up errors. If customized shields and/or conformation are used, weekly portal verification is mandatory to check the repeatability of treatment and is in any case advisable in all other cases.
- **Boost**. A postoperative dose of 16-20 Gy will be given only in cases in which surgery was not wide (margins positive or <1 mm), with margins of 2 cm on the site at risk defined by the tumor bed and marked with clips by the surgeon.
  The boost can also be given with BRT (see above) or IORT in centers where this procedure is available. The planned dose is 10-12 Gy prescribed at the isodose of 90%. The energy of the electrons used must include the target volume defined during surgery in the 90% isodose. Use of more than one field is allowed, taking care to

avoid overlapping. The planned dose limit to critical structures (peripheral nerves) is 12 Gy.

9.4 Organs at risk (OAR)

- Extremities: in treating the extremities, irradiation of the entire limb circumference must be avoided to prevent distal lymphedema, and, whenever possible, irradiation of the entire bone cortex of the long bones must be avoided to reduce the risk of fractures (24). Related to tumor site, different dose constraints will be considered (trunk, abdomen, pelvis) (25)
- Miocutaneous free flap: avoid hot spot to reduce a flap complication. In case of surgical skin flap, the use of IMRT could decrease the dose to the skin and subcutaneous tissues overlying the tumor bed. This could leave the healing properties of these "surg*ical* flaps" intact and potentially decrease the greater acute wound healing complication rate

10. RESPONSE EVALUATION

10.1 Radiological response criteria

Tumor response will have to be centrally reviewed. A web toll will be available for this purpose (sts.sarcomanet.com). Each center will be required to upload MRI and CT DICOM studies of each patient (baseline, before the second cycle and before surgery examinations) to the web tool. Reviewers (Antonella Messina and Carlo Morosi) will download the files in order to perform the radiological review. All personal information will be anonymized. Investigators' access will be protected by username and password.

Tumor assessments and determination of extent of disease will be performed at baseline, after one cycle of chemotherapy (just before starting the second) and just before surgery, with both conventional and dynamic MRI. If an MRI exam does not include a dynamic study, it will be made again. CT will be used only if MRI is contraindicated. Diffusion W MRI, DCE US and PET or PET-CT at the same time as MRI will be performed whenever possible. All exams must be performed according to the protocols described below.

Tumor response will be evaluated at each radiological assessment according to:

- Response Evaluation Criteria in Solid Tumors (revised RECIST version 1.1) (26)

Furthermore they will be exploratively evaluated:

- changes in tumor vascularisation by dynamic MRI
- changes in tumor density by contrast enhanced CT scan
- response according to Choi criteria as defined for GIST treated with imatinib applied even to MRI (27, 28)
- Suv max changes on PET scan
- changes in tumor vascularisation by DCE US

10.1.1 How to perform examinations

To perform the examination the following protocols will have to be applied:

Magnetic Resonance

- Conventional MRI
  - Pre contrast examination:

    Always: Axial T1 SE, and T2 FSE (with or without fat pre-saturation) or STIR, other sequences depending of local habits.

- Contrast examination:
  - Dynamic study: GE 3D TRless than 1 min

  GE 3D

  - TR less than 1 min (if possibile 4 sec)
  - Variable Flip angle before contrast injection (5, 10, 15, 20,25, 28)

  For 5 min

  - Diffusion study
  - Axial b50, b400, b800, b1000

Coronal or sagittal T1W sequence with fat sat

Computed Tomography

- Pre-contrast examination
- Contrast examination: to be performed using 120 ml of a conventional iodinated contrast agent, administered intravenously by an automated injector at a rate of 4 ml/sec; the contrast examination will be done in: arterial phase (delay: bolus tracking), portal phase (60 sec), delayed phase (6 min).
- Coronal mpr in delayed phase
- Tumor density will be determined by measuring CT attenuation coefficient in Hounsfield Unit (HU) by drawing a region of interest around the margin of the entire tumor, using section thickness of 5 mm in the portal and delayed phase. Two-dimensional regions of interest of the entire lesion will be drawn, and all axial sections encompassing the lesion will be included. Software will calculate semiquantitatively the mean tumor attenuation in HU defined as the average of all the pixels enclosed in the volume of interest

Double Contrast Enhanced Ultrasound

- The examinations will be performed with a sonograph using a 5MHz for deep lesions or 8.4 probe for superficial lesions
- DCE US is performed injecting, as a bolus , a whole vial of contrast medium (SonoVue) flushed immediately with 5 mL of normal saline using a peripheral venous access. The acquisition time is 3 minutes and the raw data are to be acquired. On work-station using dedicated software will be evaluated these parameters: maximum peak intensity, mean time transit, wash-in time, coefficient of the wash-in curve

10.1.2 Evaluation by RECIST Criteria

The response of each patient will be evaluated by new revised RECIST criteria after the first cycle of chemotherapy and before surgery. The original article on new RECIST criteria is published in the European Journal of Cancer (26). RECIST criteria are summarized in Table 1.

10.1.3 Evaluation by Choi Criteria, applied even to MRI

The response of each patient will be evaluated according to Choi Criteria as defined for GIST treated with imatinib (27) after the first cycle of chemotherapy and just before surgery. Choi criteria are based on changes in tumor size and density following contrast administration on CT scan. Choi criteria will be applied even to MRI assuming that changes in contrast enhancement on subtracted contrast enhanced T1 weighted sequences parallel changes in density on CT, both being markers of tumor vascularisation (28). Therefore, according to Choi criteria a radiological partial response (PR) will be defined by the presence

of a ≥10% decrease in tumor size or a ≥15% decrease in tumor density on CT scan or contrast enhancement on MRI, while progression will be qualified in case of new lesion or in case of ≥10% increase in tumor greatest maximal diameter without any criteria for PR by tumor density/contrast enhancement or ≥15% increase in tumor density/contrast enhancement without any criteria for PR by tumor size. Contrast enhancement changes on MRI will be evaluated on dynamic contrast-enhanced three-dimensional T1-weighted gradient-echo images. Choi criteria applied even to MRI are summarized in Table 1. To allow the response assessment according to Choi criteria both MRI and CT scan evaluation will be performed according to the protocol described above.

10.2 Correlation between pathological and radiological response

Discussion between radiologist and pathologist before surgical specimen section is foreseen, to identified areas of special interest according to the radiological findings (MRI, CT, PET) to be sampled in addiction to standard the axial section.

10.3 Pathological response criteria

Pathologic assessment of the surgical specimen.

All the surgical specimens should be sampled according to a standardized protocol (29). The intact surgical specimen should be oriented, in order to assess margins, ideally in the presence of the surgeon. Size of the specimen and tumour mass should be assessed before cutting in 3 dimensions. The neoplasm should be mapped on its largest section taking about a sample per 1 cm. The identification of the section should be performed in collaboration with the radiologist. In addition, all the macroscopically distinct areas (i.e., solid, cystic, hemorrhagic, necrotic) should be described and separately sampled. The result should reflect the average mapping of the whole tumour mass. Margins should be inked and separately sampled. Distance between tumor and free margins should be measured. The number of blocks of neoplasia taken and evaluated must be specified. Unfixed snap frozen samples should be taken and stored.

On histology the following characteristics of the post-treated tumour mass should be assessed:

- Percentage of residual viable tumour (RVT),
- Mitotic index
- Percentages of the other components of the mass: i.e. necrosis, hemorrhage, sclerosis, sclerohyalinosis, fibrohistiocytic reaction with haemosiderin, myxoid component, or any other observed micro/macroscopic changes.

The presence of a cystic component needs to be addressed separately as it may affect the estimation of tissue response to the treatment.

In order to achieve a more objective estimation of the different pattern of tissue response an advanced computer-aided morphometric approach will be experimented.

In the case of round cell / myxoid liposarcoma the following features should be also assessed:

- The percentages of round cell / classic component
- The amount of lipogenic differentiation as expressed by presence of lipoblasts and/or mature adipocytes.
- The presence of damage of blood vessels

11. DATA MONITORING

This study is a collaboration between multi-institutional sarcoma groups: Italian Sarcoma Group (ISG), Spanish Sarcoma Group (GEIS), French Sarcoma Group (FSG). Each group will be responsible for organizing a monitoring program of their participating institutions according to GCP. On site visits will be made at regular intervals throughout the trial as required.

11.1 Aim of monitoring

The purpose of these visits is:

to verify that the rights and well-being of human subjects are protected

to verify accuracy, completion and validity of reported trial data from the source documents

to evaluate the conduct of the trial within the institution with regard to compliance with the currently approved protocol, GCP and with the applicable regulatory requirements

11.2 Notice of monitoring

The Trials Center will give the responsible investigator adequate notice of the monitoring visit to allow adequate time, space and staff for these visits.

ISG: The ISG Clinical Trial Center at the Isituto Ortopedico Rizzoli, Bologna will be responsible for the monitoring of the participating Italian centers.

List of patients and CRFs of the different Centers undergoing monitoring will be supplied by the central Data Center.

After each monitoring visit a detailed report will be sent to the responsible investigator of the Center and to the Central data center.

ISG Clinical Trial Center

Istituto Ortopedico Rizzoli

Via di Barbiano 1/10

40136 Bologna

Tel 051 6366470

FAX 051 6366627

emanuela.marchesi@ior.it

12. TRANSLATIONAL RESEARCH

Frozen material as well as formalin-fixed paraffin-embedded material will be made available for translational research provided specific IRB is obtained. Areas of research will include identification and validation of the potential predictive markers for each histologic subgroups and attention will be posed on the analysis of the immune contexture and on its interaction with the different chemotherapy regimens. Investigation of potential novel therapeutic targets will be also one of the aims of the transitional research (TR) program. Genomic, epigenomic and proteomic approaches will be used. Specific technical platforms will be

selected within the collaborative network of the ISG, GEIS and GSF-GETO on the basis of the scientific questions to be answered. Molecular techniques will be applied whenever relevant, including expression profiling and ex vivo investigations of drug sensitivity. Molecular analysis of methylation of MGMT promoter will be performed on those specimen belonging to patients treated with alkylating agents to assess its predictive value.

TR program will include storage of paraffin embedded material as well frozen material both from pre and post chemotherapy specimen. Specifically, each center will send one formalin-fixed paraffin embedded (FFPE) representative block to Institute Bergonie, Bordeaux, to the attention of Dr. Fredric Chibon. This material will be sent by standard shipment at room temperature. Frozen material (at least one core biopsy or 50-100 mg of tumor tissue) will also be centralized in Bordeaux. Shipment will be organized 1 to three times/year. Extracted DNA/RNA will be sent to Oxford Gene Technology.
TR program will focus of prospective validation of the CINSARC prognostic signature. In this perspective, the gene expression profile of pre and post therapy tumors will be also interrogated to dissect any modification/induction of immune-related genes. As well as on identification of new prognostic/predictive signatures. Specific genomic and/or expression profiles will also be investigated in each of the five histotypes if numerous enough.
Whenever possible cases will be used for the construction of a Tissue MicroArray (TMA) that will be generated sampling both pre and post neoadjuvant therapy material. Concerning the pre therapy material. At best, two 1 mm cores will be punched from the FFPE block; concerning the surgical specimen the sampling will be done, whenever possible, as follow: three 1 mm cores from the highest grade viable tumor; one 1 mm core from the lowest grade viable tumor; two 1 mm core from the tumor edge (1 mm from tumor border); two 1 mm core from the area showing morphologically highest lymphocyte infiltrate; one 1 mm core from distant non-neoplastic tissue (preferable: 1-skin, 2-hypodermis, 3-skeletal muscle, 4-other connective tissue)

TMA will be used for testing immunohistochemically candidate markers (see below) in the perspective of investigating the main mechanisms of tumor progression, namely:

-Immune contexture

Tumor microenvironment, which includes stromal and immune cells, has a major role in cancer and the avoidance of immune recognition is a recently recognized cancer hallmark (Hanahan D et al, Cell, 2011;144(5):646-74). Immune contexture at the tumor site has a prognostic significance in many human tumor (Kirilovsky A et al, Int Immunol. 2016 Apr 27. pii: dxw021) and, importantly, it has been shown that the presence of adaptive immune infiltration is predictive for response to chemotherapy (Denkert C et al. JCO 2010;28:105-113 Denkert C J Clin Oncol. 2015 Mar 20;33(9):983-91, Halama N Cancer Res. 2011 Sep 1;71(17):5670-7). Different regimens of chemotherapy can elicit immune response either by inducing an immunogenic death of cancer cells or by the subversion of immunosuppressive mechanisms (Pol, et al Oncoimmunology. 2015 Mar 2;4(4):e1008866. 2015,). As concern soft tissue sarcoma, new evidences are now accumulating, testifying a possible role of the immune system in the natural evolution of these diseases and in modulating the response to targeted and conventional therapy (Sharma, Clin Cancr Res, 2013 19(17),4843-53, Lim J, Clin Cancer Res. 2015 Nov 1;21(21):4753-9.; Stacchiotti, Clin Cancer Res. 2016 Feb 15;22(4):837-46; Tazzari, J of Investigative. Dermatol, in press; Burgess M, Curr Oncol Rep. 2015 Nov;17(11):52). We therefore propose a deeply and accurate analysis of the immune contexture pre and post therapy with the aim of assessing:

1. a potential prognostic and predictive value of immune infiltrating cells in pre therapy tumors
2. the effect of therapy on modulating the density and nature of immune infiltration, and its relationship with clinical outcomes
   the effect of histotype on immune contexture

In brief TMA will be used for checking the immune infiltrating, which will be first characterized by IHC for the presence of T, B and NK cells by IHC staging for CD3,CD20,and CD57 and characterize for the myeloid cell infiltration by staining specific for CD14. Being positive for the above described markers, further characterization will include the assessment of the nature the infiltrating T cells performing staining for Foxp3 , detecting regulatory T cells (Treg), CD4 and CD8. Myeloid cells will be further defined by checking the staining for CD68 and CD163.
Quantitative evaluation of the immune reactivity by digital pathology will be performed scanning slides (scanner Aperio AT2, Leica Biosystems) with a 20x/0.75 NA Plan Apo ojective (Olympus), images will be captured at 40x (20x with 2x magnification changer) with a resolution of 0.25 µm/pixel. Standard pyramid tiled tagged images file format (TIFF) will be obtained and visualized with eSlide Manager (Leica Biosystems) and, after Genie software (Leica Biosystems) training – in order to remove haemosiderin signal – and algorithm optimization, analysis will performed with Cytoplasmic analysis tool (Aperio, Leica Biosystems).

These samples will be send to
Dr. Paola Collini
Struttura semplice di Patologia dei tessuti molli e dell'osso e diagnostica generale e dell'età evolutiva,
Fondazione IRCCS Istituto Nazionale dei Tumori,
via Venezian 1,
20133 Milano, Italy
and will be stored under her responsability

- Resistance
The ATP-binding cassette trans-membrane protein pumps (ABC superfamily) are involved in multidrug resistance cellular mechanisms and are responsible for cancer treatment failure. *Multidrug Resistance-Associated Protein 1 (*MRP1) is an efflux pump related with a wider range of substrate than other pumps as Pgp. These proteins have been described as prognostic factors in some tumors.
To our best knowledge, there is very scarce information about the prognostic relevance of multidrug resistance (P-gp and MRP1 among others) in STS and there are no publications regarding MRP1 as biomarker with prognostic role in STS. Most of the published information is related to retrospective series and provide information on different expression in distinct histologic subtypes (30-34).
Recently, the ISG-GEIS has provided valuable information of MRP1 and its prognostic relevance for Relapse Free Survival in 75 high risk localized STS (34). In this high risk population, MRP1 immunostaining was a significant prognostic factor for RFS ($p = 0.04$) and in the multivariate analysis was the only significant variable with HR 2.53 (1.04-6.20) with $p = 0.042$. On the other hand, the expression of *MRP1* evaluated by quantitative RT-PCR from RNA also showed the same trends toward a worse RFS if it was overexpressed (above median value) although did not reach statistical significance.

Here we propose to validate prospectively these findings.

Analysis will be performed on paraffin embedded material from surgical biopsy. To this aim each center will send to Son Espases Hospital, Palma de Mallorca, to the attention of dr. Javier Martin Broto three unstained slices of paraffin embedded blocks (stemming from diagnostic time) of 3-4 µm thick in specific slides.

- Cell cycle regulatory proteins

In the last years, the discovery of protein-protein interactions has contributed significantly on the comprehension of biological pathways, protein-networks, and signaling hubs that play a relevant role in cancer development and progression.

In the case of p53, this knowledge is beginning to be translated into cancer patient stratification that complements the advances already being made using gene mutation status and transcription profiling to predict therapeutic responses. One of the key aims of translational biomedicine is to link this vast information on p53 interactors into clinically relevant cancer therapeutics. In a recent paper of ours Piccinin et al. (Cancer cell, 2012) we made the effort to bridge this gap by demonstrating that the mesenchymal signaling protein Twist1 forms a clinically relevant inhibitory p53 interaction in human sarcomas.

Numerous evidence have shown that *MDM2* plays an important role in the inactivation of the p53 response in sarcomas and that the ratio of spliced MDM4 full-length and smaller isoforms provides an important information in terms of survival rates, better than the mutation status of p53. Therefore, there is a significant potential to stratify patients in sarcoma clinical trials that might benefit from MDM2 or MDM4 targeted therapeutics.

Despite this enormous promise in targeting MDM2 to reactivate p53, human sarcomas are a very heterogeneous group of cancers arising from mesenchymal tissues within fat, muscle, peripheral nerves, fibrous tissue, or bone, and the wt-p53 alleles are not always retained. Even in sarcomas with wt-p53 alleles, MDM2 is not always the dominantly expressed p53 suppressor, and identification of such clinically relevant p53-inhibitors is a prime goal. We have demonstrated that Twist1, by interacting and inhibiting p53 in sarcoma cells, exerts such a dominant pro-oncogenic function.

Thus, Twist1 stands as a novel elements for a better stratification of sarcoma patients. At the same time, targeting the Twist1-p53 complex stands as an unprecedented, clinically relevant approach for reactivating p53, but it also complements the existing potential to target synergistically other p53 inhibitory pathways.

Here we propose to address these issues by:

1) Investigating the role of Twist1, in combination with the assessment of the expression and gene status of p53 (p53, p63, p73) and MDM2 protein families (MDM2 and MDMX), as diagnostic and prognostic marker in selected sarcoma histotypes (Unità Operativa Complessa di Anatomia Patologica, Treviso)

- Angiogenesis

Immunohistochemical analysis of angiogenesis markers such as VEGF, VEGFR, HIG, FGF, HGF and PDGF would be also performed, as growth and dissemination of sarcomas strongly depends upon neo-angiogenesis.

- Metastasis/invasion markers

The possible role of molecules know to be involved in the mechanism of cell-cell and cell-

matrix interactions, such as LFA-1 (leukocyte function associated antigen-1; CD11a), HCAM (CD44), VLA-2 (CD49b), ICAM-1 (CD54), NCAM (CD56) and LECAM-1 (CD62L) and CD86). will be investigated.

- Senescence markers

Cellular senescence is an irreversible growth-arrest mechanism that protects the cell from hyperproliferative signals and various forms of stress such as DNA damage. Different studies have shown, in animal models, that senescence induced by DNA damage is a tumor suppressor mechanism by itself. Moreover, DNA damage is one of the mechanisms used by some cancer treatments, like radiotherapy or some chemotherapies (adriamycin, cisplatin). On this basis an evaluation of senescence biomarkers will be performed.

13. PUBLICATION POLICY

The final publication of the trial results will be written by the Study Chairman on the basis of the final analysis performed at IST Genova. The first author will be the Study Chairman. Investigator who has entered at least 5% of the eligible patients will be included as a co-author, up to the maximum number of authors accepted by the journal. Co-authors will also be a representative of diagnostic review panels (pathology and radiology), the statistician, the data manager and at least one representative of each other Group that has entered patients in the study. The sequence of the authors in publication will be the Study Chairman, and subsequent authors according to the number of patients entered in the study. When applicable, all remaining investigators who have entered at least 1 eligible patient will be listed at the end as collaborators. The manuscript will also include an appropriate acknowledgment section, mentioning all groups that have contributed to the study, as well as supporting bodies (sponsors…). All publications (papers, abstracts, presentations…) including data from the present trial will be submitted for review to the Steering Committee and to all co-authors prior to submission. Publications on secondary objectives as well as on retrospective unplanned analyses, that will be performed by any of the collaborating center/group with the data issued from this study, will be allowed, upon approval by the Trial Steering Committee. The Study Chairman and Steering Committee must approve all publications, abstracts and presentations of data pertaining to patients included in this study. This is applicable to any individual patient registered/randomized in the trial, or any subgroup of these.

14. PATIENTS RANDOMIZATION PROCEDURE

Fully eligible patients will be asked to provide the formal informed consent to the study. The aims and methods of the study will be thoroughly discussed with the eligible patients who will be provided with:

1. the study information brochure approved by the local competent authority reporting in a simple language the study aims and methods, the surgical/clinical procedures, the associated risks and/or potential side effects, their right to withdraw at any time without any specific reason,
2. the informed consent form approved by the local competent authority and invited to participate in the study.

Only consenting patients (i.e., those who have signed the informed consent form) will then undergo all the pre-randomization screening tests. Consenting eligible patient from Group 1 and 3 will then be assigned at random to the standard (epirubicine + ifosfamide) or the

histotype-tailored chemotherapy group according to a randomization list generated to fulfill the planned allocation criteria of patients belonging to Group 1 and Group 3 to the Arm A (standard chemotherapy based on full-dose epirubicine + ifosfamide) and Arm B (histotype-tailored chemotherapy: B_1-B_5). Group 2 patients will all be allocated to Arm A.

15. ADVERSE EVENTS

Adverse events
An adverse event for the purposes of this protocol is the appearance of (or worsening of any pre-existing) undesirable sign(s), symptom(s), or medical condition(s) occurring after signing the informed consent even if the event is not considered to be related to chemotherapy. Adverse events will be assessed according to the Common Toxicity Criteria for Adverse Events (NCI - CTC) version 4.03.

As far as possible, each adverse event should be evaluated to determine:
1.The CTCAE grade 1-4
2.Its relationship to each study drug (suspected/not suspected)
3.Its duration (start and end dates or if continuing at final exam)
4.Action taken (no action taken; study drug dosage adjusted/temporarily interrupted; study drug permanently discontinued due to this adverse event; concomitant medication taken; non-drug therapy given; hospitalization/prolonged hospitalization)
5.Whether it is serious, where a serious adverse event (SAE) is defined as one which:
 Is fatal or life-threatening
 Results in persistent or significant disability/incapacity
 Constitutes a congenital anomaly/birth defect
 Requires inpatient hospitalization or prolongation of existing hospitalization, unless hospitalization is for:
 Routine treatment or monitoring of the studied indication, not associated with any deterioration in condition
 Elective or pre-planned treatment for a pre-existing condition that is unrelated to the indication under study and has not worsened since signing the informed consent
 Treatment on an emergency outpatient basis for an event not fulfilling any of the definitions of a SAE given above and not resulting in hospital admission
 Social reasons and respite care in the absence of any deterioration in the patient's general condition
 Is medically significant, i.e., defined as an event that jeopardizes the patient or may require medical or surgical intervention to prevent one of the outcomes listed above

Unlike routine safety assessments, SAEs are monitored continuously and have special reporting requirements

All adverse events should be treated appropriately. Such treatment may include changes in study drug treatment including possible interruption or discontinuation, starting or stopping concomitant treatments, changes in the frequency or nature of assessments, hospitalization, or any other medically required intervention.

All adverse events that occur between the first study-related procedure and 3 months post administration of the last dose of study drug (or after this date if the investigator feels the

event is related to the study drug) must be recorded by Investigators into the CRF. Those meeting the definition of Serious Adverse Event (SAE) related or not to the Study Treatment and reports of drug exposure during pregnancy will be forwarded by the investigators within 24 hours of becoming aware of a report which contains the minimum data elements for a valid case using the Serious Adverse Event Form or Pregnancy Form (Appendix 1 and 2). All forms must be dated and signed by Principal Investigators or one of his/her authorized staff members. The date of receipt of the SAE or Pregnancy report will be considered "Day 0" for regulatory reporting purposes. Any additional details required to Investigators must be documented and transmitted by fax or e-mail within 24 hours from the request.

Italian investigators participating through ISG group must transmit Serious Adverse Event Form and Pregnancy Form to:

Centro Clinical Trials
Fax: 010354103
e-mail: clinicaltrials@istge.it

European investigators (other than the Italians) participating through Country specific Sarcoma Groups (e.g., GEIS, GSF-GETO) must follow National/local instructions for reporting of Serious Adverse Event and Pregnancy.

15. INDEPENDENT DATA MONITORING COMMITTEE

15.1 Introduction

An Independent Data Monitoring Committee (IDMC) will assess the progress of the trial, the safety data and the critical efficacy endpoints. The members of the IDMC serve in an individual capacity and provide their expertise and recommendations to the Sponsor (or representative) and Steering Committee. The IDMC will review cumulative study data to evaluate the safety, the study conduct as well as the scientific validity and the data integrity of the study.

15.2 Composition of the IDMC

The Committee will be composed of 3 independent international experts:

- Medical oncologist specialized in the field of Soft Tissue Sarcoma
- Surgeon specialized in the field of Soft Tissue Sarcoma
- Methodologist

Independence of the members is essential to ensure that IDMC members are objective and capable of unbiased assessment of the study's safety and efficacy data. Members of the IDMC must not have a direct interest (financial or intellectual) in knowing or influencing the trial outcome. IDMC members must disclose all pharmaceutical companies, biotech, CROs in which they have financial interest. The IDMC Chairman will be the primary contact person. All members of the IDMC will be regarded as having equal authority and will have to reach a global decision after each meeting. If one of the members misses more than 3 IDMC meetings, the member should be replaced.

15.2 Roles of the IDMC

The role of the IDMC will be:

- to review accrual rate

- to examine the Development Safety Update Report (DSUR)

The yearly interim futility analyses will be reported to the IDMC:

- these interim analyses will remain confidential
- on the basis of these analyses, the IDMC will recommend whether the study can continue, or whether it should be changed or terminated prematurely.

15.3 IDMC Communication

Meeting Minutes

The meetings will consist of an open and a closed session as appropriate. The IDMC meetings will be documented on meeting minutes. The IDMC Chairman will prepare the draft meeting minutes and forward to the IDMC members for review within one week following the IDMC meeting. Draft minutes will be distributed to all meeting attendees for review and comment. Minutes from the closed session will remain with the IDMC chairman and not be forwarded to the sponsor until end of the trial.

IDMC reports will be sent to the Sponsor and the Steering Committee following IDMC meetings. IDMC reports will not contain confidential information from the closed meeting except conclusions and recommendations to the sponsor.

Post-meeting Sponsor's decision

Following receipt of the IDMC report, the Sponsor and the Steering Committee will evaluate the IDMC recommendations and take the final decision. A brief teleconference will be held if needed between the IDMC Chair and the Sponsor representatives to discuss the recommendations of the IDMC.

15.4 **Competent authority and Ethic Committee**

All IDMC meeting reports will be archived by the Sponsor. They will be sent to CA and IRB/Ethic Committee when requested.

15.5 Confidentiality

All data provided to the IDMC and all deliberations of the IDMC will be privileged and confidential. The IDMC will agree to use this information to accomplish its responsibilities and will not use it for other purposes without written consent from the Sponsor. No communication of the deliberations or recommendations of the IDMC, either written or oral, will occur except as required for the IDMC to fulfil its responsibilities. Individual IDMC members must not have direct communication regarding the study outside the IDMC (including, but not limited to the investigators, IRB/EC, regulatory agencies, or sponsor) except as authorized by the IDMC.

TABLE 1: RECIST criteria and Choi criteria applied even to MRI

Response	RECIST	CHOI
CR	- Disappearance of all lesions.<br>- No new lesions.	- Disappearance of all lesions.<br>- No new lesions.
PR	- A ≥ 30% decrease in the sum of greatest diameters.<br>- No new lesions.	- A ≥ 10% decrease in the greatest maximal diameter or a ≥ 15% decrease in tumor density (HU) / contrast enhancement on CT / MRI.<br>- No new lesions.
SD	- Does not met criteria for CR, PR or PD.	- Does not met criteria for CR, PR or PD.
PD	- A ≥ 20% increase in the sum of greatest diameters.<br>- New lesion	- A ≥ 10% increase in the greatest maximal diameter and does not meet criteria for PR by tumor density (HU) / contrast enhancement on CT / MRI or ≥ 15% increase in tumor density (HU) / contrast enhancement on CT / MRI and does not meet the criteria for PR by tumor size.<br>- New lesion.<br>- New intratumoral nodule or increase in the size of existing intratumoral nodule.

16. REFERENCES

1) Delaney TF. Adjuvant therapy for adult patients with soft tissue sarcomas. *Oncology* 1991; 5: 105-118.
2) Potter DA et al. Pattern of recurrence in patients with high-grade soft tissue sarcomas. *J Clin Oncol* 1985; 3: 353-366.
3) Sarcoma Meta-Analysis Collaboration. Adjuvant chemotherapy for localized resectable soft-tissue sarcoma of adults: meta-analysis of individual data. *Lancet* 1997; 350: 1647-1654.
4) *Pervaiz N, Colterjohn N, Farrokhyar F et al. A systematic meta-analysis of randomized controlled trials of adjuvant chemotherapy for localized resectable soft-tissue sarcoma. Cancer 2008; 113 (3): 573-581*
5) Antman K, Crowley J, Balcerzak SP, et al. An Intergroup phase III randomized study of doxorubicin and dacarbazine with or without ifosfamide and mesna in advanced soft tissue and bone sarcoma. *J Clin Oncol* 1993; 11: 1276-1285.
6) Edmonson JH, Ryan LM, Blum RH, et al. Randomized comparison of doxorubicin alone versus ifosfamide plus doxorubicin or mitomycin, doxorubicin, and cisplatin against advanced soft tissue sarcomas. *J Clin Oncol* 1993; 11: 1269-1275.
7) Santoro A, Tursz T, Mouridsen H, et al. Doxorubicin versus CYVADIC versus doxorubicin plus ifosfamide in first-line treatment of advanced soft tissue sarcomas: a randomized study of the European Organization for Research and Treatment of Cancer Soft Tissue and Bone Sarcoam Group. *J Clin Oncol* 1995; 13: 1537-1545.
8) Frustaci S, Gherlinzoni F, De Paoli A, et al. Adjuvant chemotherapy for adult soft tissue sarcomas of the extremities and girdles: results of the Italian randomized cooperative trial. *J Clin Oncol* 2001; 19: 1238-1247.
9) Gronchi A, Frustaci S, Mercuri M, et al. Localized high-risk soft tissue sarcoma (STS) of the extremities and trunk wall n adults: Three versus five cycles of full-dose anthracyclin and ifosfamide adjuvant chemotherapy: A phase III randomized trial from the Italian Sarcoma Group (ISG) and Spanish Sarcoma Group (GEIS). J Clin Oncol 2010, 28:15 s (abstr 10003).
10) Le Cesne A, Antoine E, Spielman M, et al. High dose Ifosfamide: circumvention of resi stance to standard dose ifosfamide in advanced soft tissue sarcoma, *J Clin Oncol* 1995; 13: 1600-1608.
11) Penel N, Bui BN, Bay JO, et al. Phase II trial of weekly paclitaxel for unresectable angiosarcoma: the ANGIOTAX Study. J Clin Oncol. 2008 Nov 10; 26 (32): 5269-5274.
12) Duffaud F, Pautier P, Bui Nguyen B,et al. A pooled analysis of the final results of the two randomized phase II studies comparing gemcitabine (G) vs gemcitabine + docetaxel (G + D) in patients with metastatic/relapsed leyomiosarcoma (LMS). *Ann Oncol 2010; 21 suppl 8: abs 13450.*
13) Garcia Del Muro, J. Fra, A. Lopez Pousa, J. Maurel, et al Randomized phase II study of dacarbazine plus gemcitabine versus DTIC alone in patients with advanced soft tissue sarcoma: A Spanish Group for Research on Sarcomas (GEIS) study. *J Clin Oncol 2009 27:15s, (suppl; abstr 10529).*
14) Maki RG, Wathen JK, Patel SR, et al. Randomized phase II study of gemcitabine and docetaxel compared with gemcitabine alone in patients with metastatic soft tissue sarcomas: results of sarcoma alliance for research through collaboration study 002 J Clin Oncol. 2007 Jul 1;25(19):2755-63. Erratum in: J Clin Oncol 2007 Aug 20; 25 (24): 3790.

15) Grosso F, Jones RL, Demetri GD, et al. Eficacy of trabectedin (ecteinascidin-743) in advanced pretreated myxoid liposarcomas: a retrospective study. Lancet Oncol 2008: 595–602.
16) Jones RL, Fisher C, Al-Muderis O, Judson IR. Differential sensitivity of liposarcoma subtypes to chemotherapy. Eur J Cancer 2005 Dec; 41(18): 2853-60.
17) Hensley ML, Blessing JA, Mannel R, Rose PG. Fixed-dose rate gemcitabine plus docetaxel as first-line therapy for metastatic uterine leiomyosarcoma: A Gynecologic Oncology Group phase II trial. Gynecol Oncol 2008; 109: 329–334
18) Casali PG, Jost L, Sleijfer S et al. Soft tissue sarcomas: ESMO clinical recommendations for diagnosis, treatment and follow-up. Ann Oncol 2009; 20 Suppl 4:132-136.
19) Fletcher C.D.M, Unni K.K, Mertens F. World Health Organization Classification of Tumours. Pathology and Genetics of Tumours of Soft Tissue and Bone. 2003.
20) Trojani M, Contesso G, Coindre JM, et al. Soft-tissue sarcomas of adults; study of pathological prognostic variables and definition of a histopathological grading system. Int J Cancer 1984; 33: 37-42.
21) Oken MM, Creech RH, Tormey DC, et al. Toxicity and response criteria of the Eastern Cooperative Oncology Group. Am J Clin Oncol 1982; 5: 649-655.
22) The Criteria Committee of the New York Heart Association, Inc: Diseases of the Heart and Blood Vessels; Nomenclature and Criteria for Diagnosis, 6th ed Boston, Little, Brown 1964.
23) Monitoring of large randomized clinical trials: a new approach with Bayesian methods. *Parmar MK, Griffiths GO, Spiegelhalter DJ, Souhami RL, Altman DG, van der Scheuren E; CHART steering committee. Lancet. 2001 Aug 4; 358 (9279): 375-81).*
24) O'Sullivan B, Int. J. Radiation Oncology Biol. Phys., Vol. 75, No. 4, pp. 1119–1124, 2009
25) Anthony M Griffin; Int. J. Radiation Oncology Biol. Phys., Vol. 67, No. 3, pp. 847–856, 2007)
26) Eisenhauer EA, Therasse P, Bogaerts J, et al: New response evaluation criteria in solid tumours: revised RECIST guideline (version 1.1). Eur J Cancer. 45:228-47, 2009).
27) Choi H, Charnsangavej C, Faria SC, at al. Correlation of computed tomography and positron emission tomography in patients with metastatic gastrointestinal stromal tumor treated at a single institution with imatinib mesylate: proposal of new computed tomography response criteria. J Clin Oncol 25:1753–1759, 2007
28) Stacchiotti S, Collini P, Messina A, et al. Tumor response assessment in high grade soft tissue sarcomas (STS): a pilot study to assess the correlation between radiological and pathological response using both RECIST and Choi's criteria. Radiology, 251:447-56, 2009
29) Rubin BP, Fletcher CD, Inwards C, et al. Protocol for the examination of specimens from patients with soft tissue tumors of intermediate malignant potential, malignant soft tissue tumors, and benign/locally aggressive and malignant bone tumors. Arch Pathol Lab Med 2006; 130:1616-1629
30) Citti A, Boldrini R, Inserra A et al. Expression of multidrug resistance-associated proteins in paediatric soft tissue sarcomas before and after chemotherapy. Int J Oncol. 2012; 41(1):117-24.
31) Oda Y, Saito T, Tateishi N et al. ATP-binding cassette superfamily transporter gene expression in human soft tissue sarcomas. Int J Cancer. 2005 May 10; 114(6):854-62.
32) Komdeur R, Molenaar WM, Zwart N. et al. Multidrug resistance proteins in primary and metastatic soft-tissue sarcomas: down-regulation of P-glycoprotein during metastatic progression. Anticancer Res. 2004; 24(1):291-5.

33) R Komdeur, B.E.C Plaat, W.T.A van der Graaf. Expression of multidrug resistance proteins, P-gp, MRP1 and LRP, in soft tissue sarcomas analysed according to their histological type and grade. Eur J Cancer 2003; 39 (7):909-16
34) J Martin-Broto, P Luna, A López-Pousa et al. CTOS oral presentation. 26-29 Oct 2011, Chicago.
