# Supplementary material for: Histopathological Response After Neoadjuvant Chemotherapy for High-Risk Soft-Tissue Sarcomas: A Secondary Analysis of a Randomized Clinical Trial
Source: JAMA Netw Open. 2025 Nov 6;8(11):e2540177. doi: 10.1001/jamanetworkopen.2025.40177 (PMC12593128; doi:10.1001/jamanetworkopen.2025.40177)
Supplement: Supplement 2. — eMethods. eFigure 1. Representation of Schelorohyalinosis eFigure 2. Distribution of the Characteristics of Histopathological Response Considered in This Study in All Evaluable Patients n=388 eTable. Distribution of Characteristics of Histopathological Response eReferences [file jamanetwopen-e2540177-s002.pdf]

Supplemental Online Content

Pasquali S, Collini P, Romagosa C, et al. Histopathological response after neoadjuvant chemotherapy for high-risk soft-tissue sarcomas: a secondary analysis of a randomized clinical trial. *JAMA Netw Open*. 2025;8(10):e2540177. doi:10.1001/jamanetworkopen.2025.40177

eMethods

eFigure 1. Representation of Schelorohyalinosis

eFigure 2. Distribution of the Characteristics of Histopathological Response Considered in This Study in All Evaluable Patients n=388

eTable. Distribution of Characteristics of Histopathological Response

eReferences

This supplemental material has been provided by the authors to give readers additional information about their work.

© 2025 Pasquali S et al. *JAMA Network Open*

eMethods

Patients

This was a pre-planned translational study of the ISG-STS 1001 clinical trial[1,2]. The ISG-STS 1001 trial was a European, investigator-initiated, multicenter prospective clinical study funded by the European Union through the EUROSARC FP7 grant (278472), sponsored by the Italian Sarcoma Group and conducted between 2011 and 2016. The trial involved 32 centers across Italy, Spain, France, and Poland. The study enrolled patients with primary, resectable STS of the extremities and trunk wall, meeting high-risk criteria. Eligible tumors were characterized by high malignancy grade (grade 3 according to the Fédération Nationale des Centres de Lutte Contre le Cancer [FNCLCC] grading system, or grade 2 with >50% necrosis on baseline imaging) as assessed by a pretreatment needle biopsy, deep location relative to the investing fascia, and a tumor diameter >5 cm at baseline assessment.

ISG-STS 1001 included both a randomized controlled trial (RCT) and a non-randomized patient cohort. The RCT was an open-label, phase 3 study in which patients were randomly assigned (1:1) to receive either three cycles of neoadjuvant anthracycline plus ifosfamide (AI) or histology-tailored (HT) chemotherapy. Eligible histologies included myxoid liposarcoma (HG-MLPS), leiomyosarcoma (LMS), synovial sarcoma (SS), malignant peripheral nerve sheath tumor (MPNST), and undifferentiated pleomorphic sarcoma (UPS). In the HT arm, chemotherapy regimens were tailored according to tumor histotype: HG-MLPS: trabectedin; LMS: gemcitabine plus dacarbazine; SS: high-dose ifosfamide; MPNST: etoposide plus ifosfamide; UPS: gemcitabine plus docetaxel. A centralized pathological review of pretreatment biopsy assessed the primary tumor histotype and grading. FNCLCC grading system was applied to all histotypes but myxoid liposarcoma. High-grade MLPS was defined as a myxoid liposarcoma with neoplastic cell density >5% in more than 5% of the bioptic specimen.

Preoperative radiotherapy (RT) was administered at the physician's discretion. However, patients with LMS or UPS selected for preoperative RT could not receive the corresponding HT chemotherapy schedules (gemcitabine plus dacarbazine, or etoposide plus ifosfamide, respectively). As a result, these patients were not randomized and were included in the non-randomized cohort. This non-randomized cohort also included patients with myxofibrosarcoma, pleomorphic liposarcoma, pleomorphic rhabdomyosarcoma, and unclassified spindle cell sarcoma. Details of the study design and chemotherapy schedules are available in previous publications and the study protocol.

Macroscopic examination of the surgical specimen

Surgical specimens were processed and sampled according to a standardized protocol [3]. In order to assess margins, the intact surgical specimen was oriented, when possible, in the presence of surgeons who performed the surgical procedure. Three dimension-size of both the whole specimen and tumor mass were

assessed. Following the Protocol recommendations, the neoplasm was mapped on a grid, taking about a sample per 1 cm of the most representative section selected in collaboration with a radiologist usually along the largest dimension, considering the pre-operative MRI scan. All the macroscopically different areas not included in the grid were also described and separately sampled. When the collaboration with a radiologist was not available or when unfeasible, the neoplastic mass was sampled taking a sample per cm along the largest diameter from the vary macroscopically different areas. Margins were inked and separately sampled.

Statistical analysis

Association between continuous variables was assessed by Spearman correlation coefficient. A Cox regression model [4] was implemented to evaluate the association between each characteristic of histopathological response and DFS. The DFS patterns were estimated using the Kaplan–Meier method [5] and the survival curves were compared using log-rank test. All statistical analyses were carried out with SAS Studio (version 5.2, SAS Institute, Inc., Cary, NC, USA) by adopting a significance level of 0.05.

Results

Distribution of characteristics of histopathological response

We observed a relevant correlation (Spearman Correlation Coefficient greater than 0.30) between necrosis and proportion of stainable tumor cells (r=-0.43; 95%CI: -0.50, -0.34), showing that patients with low proportion of stainable tumor cells r have larger amount of necrosis. A higher prevalence of sclerohyalinosis correlated with lower proportion of stainable tumor cells (r=-0.32; 95%CI: -0.41, -0.23), necrosis (r=-0.32; 95%CI: -0.41, -0.23) and sclerosis (r=-0.34; 95%CI: -0.42, -0.25). These findings characterize the histopathological response based on different selected features, which collectively represent the entire tumor lesion (100%) when summed.

© 2025 Pasquali S et al. *JAMA Network Open*

eFigure 1. Representation of schelorohyalinosis

A single tumor stainable cells entrapped   in the sclerohyalinosis is visible in the figures at different magnifications.

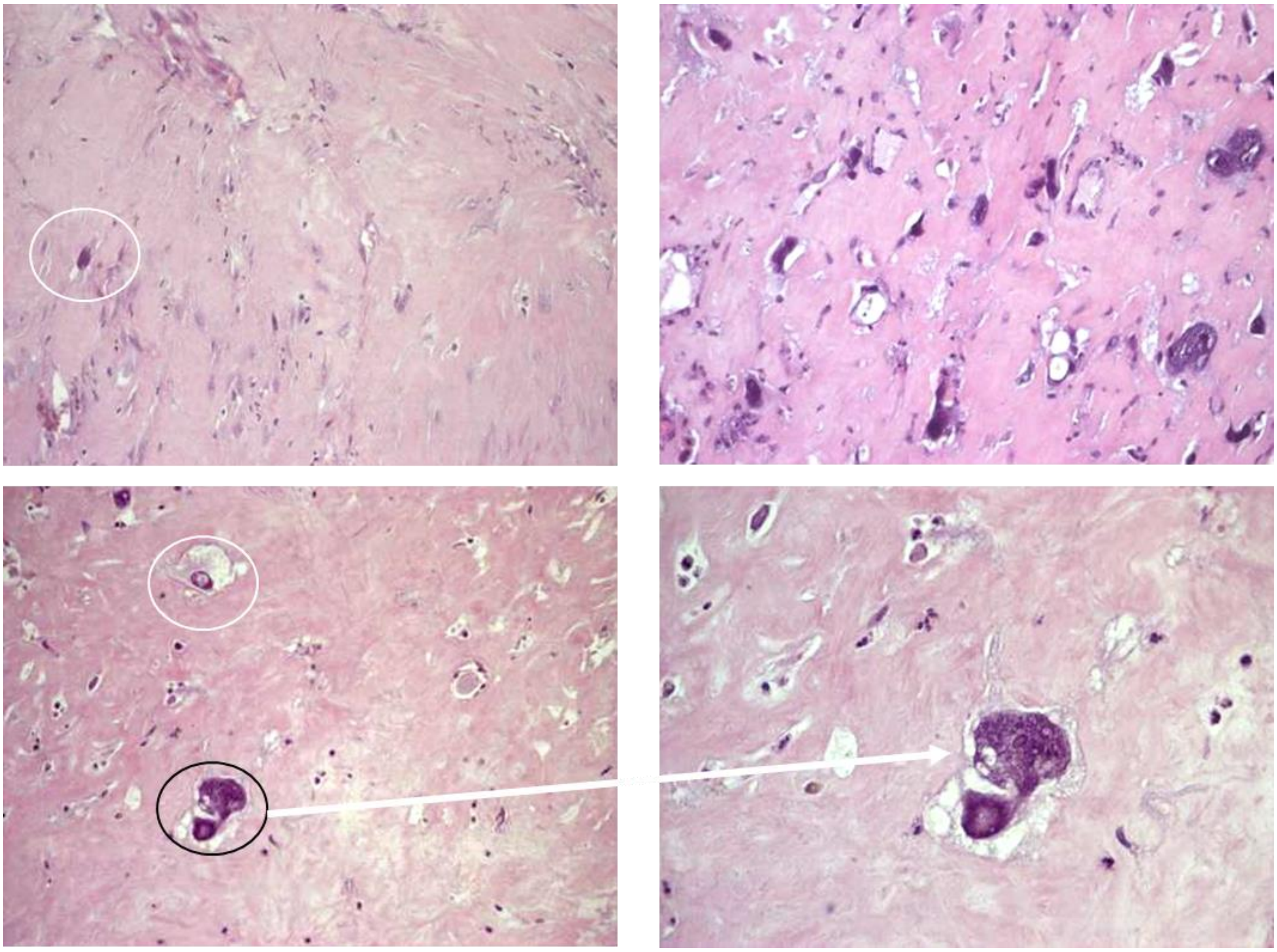

© 2025 Pasquali S et al. *JAMA Network Open*

eFigure 2. Distribution of the characteristics of histopathological response considered in this study in all evaluable patients n=388

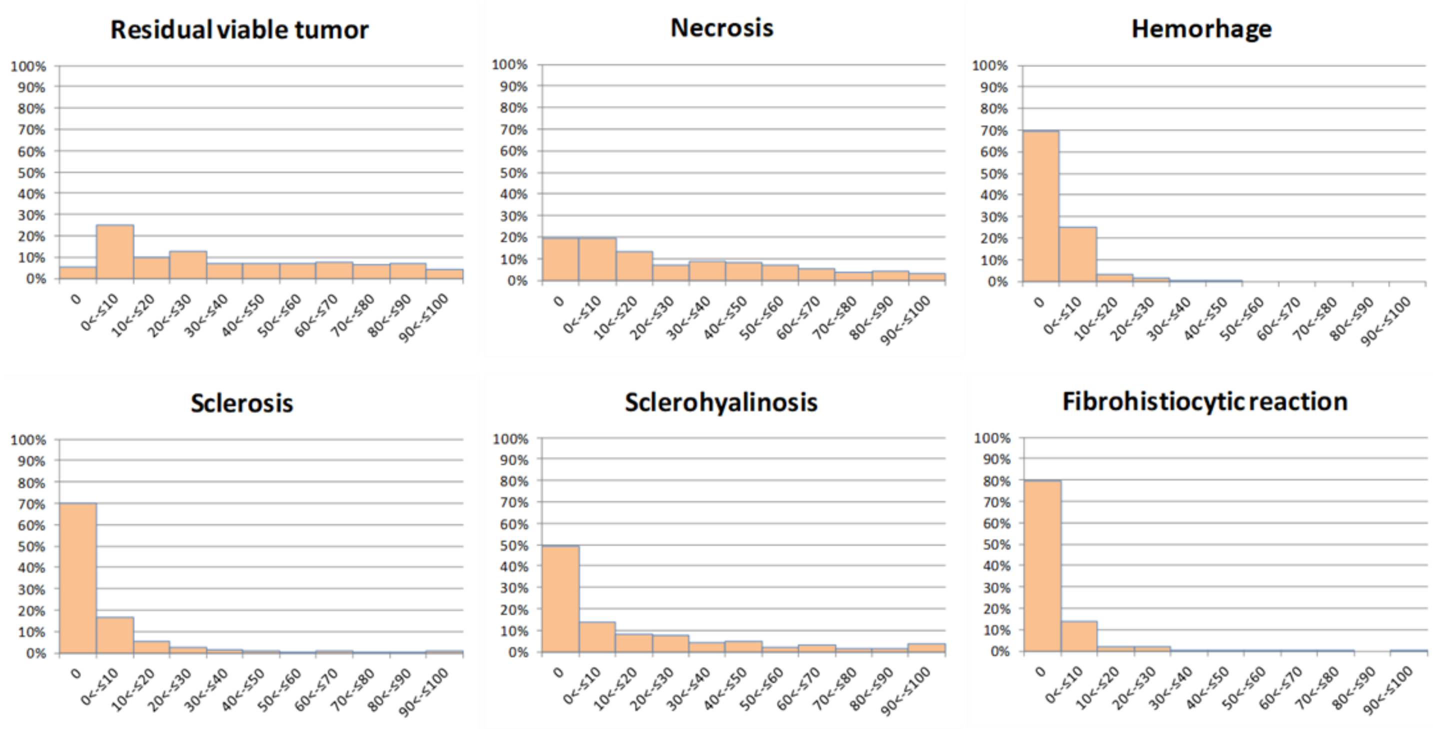

© 2025 Pasquali S et al. *JAMA Network Open*

eTable. Distribution of characteristics of histopathological response

Characteristics of histopathological response	No.	Min	25th	Median	75th	Max	IQR
Residual stainable tumor cells (%)	388	0	10	30	65	100	55
Necrosis (%)	388	0	5	20	50	100	45
Hemorhage (%)	388	0	0	0	5	50	5
Fibrohistiocytic reaction with haemosiderin (%)	388	0	0	0	0	96	0
Fibrosis / Sclerosis (%)	388	0	0	0	5	100	5
Sclerohyalinosis (%)	388	0	0	1.5	30	100	30

© 2025 Pasquali S et al. *JAMA Network Open*

eReferences

1. Gronchi A, Ferrari S, Quagliuolo V, et al. Neoadjuvant chemotherapy in high-risk soft tissue sarcomas: a randomised clinical trial from the Italian Sarcoma Group, the Spanish Sarcoma Group (GEIS), the Italian French Group (FSG) and the the Polish Sarcoma Group (PSG). . *Lancet Oncol*. 2017;
2. Gronchi A, Palmerini E, Quagliuolo V, et al. Neoadjuvant Chemotherapy in High-Risk Soft Tissue Sarcomas: Final Results of a Randomized Trial From Italian (ISG), Spanish (GEIS), French (FSG), and Polish (PSG) Sarcoma Groups. *J Clin Oncol*. Jul 1 2020;38(19):2178-2186. doi:10.1200/JCO.19.03289
3. Rubin BP, Fletcher CD, Inwards C, et al. Protocol for the examination of specimens from patients with soft tissue tumors of intermediate malignant potential, malignant soft tissue tumors, and benign/locally aggressive and malignant bone tumors. *Arch Pathol Lab Med*. Nov 2006;130(11):1616-29. doi:10.5858/2006-130-1616-PFTEOS
4. Cox RD. Regression models and life tables. *J R Stat Soc B* 1972;34:187–220.
5. Kaplan EL, Meier P. Nonparametric estimation from incomplete observations. *J Am Stat Assoc* 1958;53:457–481.
