# Supplementary material for: Histopathological Response After Neoadjuvant Chemotherapy for High-Risk Soft-Tissue Sarcomas: A Secondary Analysis of a Randomized Clinical Trial
Source: JAMA Netw Open. 2025 Nov 6;8(11):e2540177. doi: 10.1001/jamanetworkopen.2025.40177 (PMC12593128; doi:10.1001/jamanetworkopen.2025.40177)
Supplement: Supplement 3. — Data Sharing Statement [file jamanetwopen-e2540177-s003.pdf]

Data Sharing Statement

Pasquali. Histopathological Response After Neoadjuvant Chemotherapy for High-Risk Soft-Tissue Sarcomas. *JAMA Netw Open*. Published October 30, 2025.
doi:10.1001/jamanetworkopen.2025.40177

Data
Additional Information: Localized High-Risk Soft Tissue Sarcomas Of The Extremities And Trunk Wall In Adults: An Integrating Approach Comprising Standard Vs Histotype-Tailored Neoadjuvant Chemotherapy. Clinicaltrial.gov ID:NCT01710176
Data available: Yes
Data types: Deidentified participant data
How to access data: Study data could be requested to the corresponding author (sandro.pasquali@istitutotumori.mi.it) upon reasonable requests.
When available: With publication

Supporting Documents
Document types: None

Additional Information
Who can access the data: Data will be made available researchers whose proposed use of the data has been approved
Types of analyses: Data will be made available for specified purposes.
Mechanisms of data availability: Data will be made available after a data transfer agreement.
